# Interindividual Age-Independent Differences in Human CX43 Impact Ventricular Arrhythmic Risk

**DOI:** 10.34133/research.0254

**Published:** 2023-11-15

**Authors:** Laura García-Mendívil, María Pérez-Zabalza, Antoni Oliver-Gelabert, José María Vallejo-Gil, Javier Fañanás-Mastral, Manuel Vázquez-Sancho, Javier André Bellido-Morales, Alexánder Sebastián Vaca-Núñez, Carlos Ballester-Cuenca, Emiliano Diez, Laura Ordovás, Esther Pueyo

**Affiliations:** ^1^ Biomedical Signal Interpretation and Computational Simulation group (BSICoS), Aragón Institute of Engineering Research, University of Zaragoza, Zaragoza 50018, Spain.; ^2^ BSICoS, Instituto de Investigación Sanitaria Aragón (IISA), Zaragoza 50018, Spain.; ^3^ Centro Universitario de la Defensa (CUD), Zaragoza 50090, Spain.; ^4^ Department of Cardiovascular Surgery, University Hospital Miguel Servet, Zaragoza 50009, Spain.; ^5^ Institute of Experimental Medicine and Biology of Cuyo (IMBECU), CONICET, Mendoza 5500, Argentina.; ^6^ Fundación Agencia Aragonesa para la Investigación y el Desarrollo (ARAID), Zaragoza 50018, Spain.; ^7^ Biomedical Research Networking Center in Bioengineering, Biomaterials and Nanomedicine (CIBER-BBN), Zaragoza 50018, Spain.

## Abstract

Connexin 43 (CX43) is one of the major components of gap junctions, the structures responsible for the intercellular communication and transmission of the electrical impulse in the left ventricle. There is limited information on the histological changes of CX43 with age and their effect on electrophysiology, especially in humans. Here, we analyzed left ventricular biopsies from living donors starting at midlife to characterize age-related CX43 remodeling. We assessed its quantity, degree of lateralization, and spatial heterogeneity together with fibrotic deposition. We observed no significant age-related remodeling of CX43. Only spatial heterogeneity increased slightly with age, and this increase was better explained by biological age than by chronological age. Importantly, we found that CX43 features varied considerably among individuals in our population with no relevant relationship to age or fibrosis content, in contrast to animal species. We used our experimental results to feed computational models of human ventricular electrophysiology and to assess the effects of interindividual differences in specific features of CX43 and fibrosis on conduction velocity, action potential duration, and arrhythmogenicity. We found that larger amounts of fibrosis were associated with the highest arrhythmic risk, with this risk being increased when fibrosis deposition was combined with a reduction in CX43 amount and/or with an increase in CX43 spatial heterogeneity. These mechanisms underlying high arrhythmic risk in some individuals were not associated with age in our study population. In conclusion, our data rule out CX43 remodeling as an age-related arrhythmic substrate in the population beyond midlife, but highlight its potential as a proarrhythmic factor at the individual level, especially when combined with increased fibrosis.

## Introduction

Gap junctions, formed predominantly by connexin 43 (CX43) in the ventricular myocardium, are intercellular communication structures found in the heart, primarily at the intercalated discs connecting adjacent cardiomyocytes longitudinally. At lower densities, gap junctions are also found at the lateral borders of cardiomyocytes. Gap junctions connect the cytoplasm of adjacent cells, allowing the exchange of ions and small intracellular molecules between them that lead to electrical and metabolic synchronization. This is particularly important in heart tissue, where they mediate the electrical cell coupling required for the transmission of the electrical excitation. The impulse normally propagates at a higher conduction velocity (CV) along the longitudinal axis of cardiomyocytes than in the transverse one [1].

Altered Cx43 expression and/or distribution can affect ventricular electrical coupling and can lead to impulse conduction abnormalities and arrhythmias, particularly when combined with additional structural and functional changes associated with pathological remodeling or aging. In humans, altered CX43 patterns have been reported in atrial fibrillation, heart failure, dilated cardiomyopathy, or left ventricular hypertrophy [2–4]. Animal models with reduced Cx43 expression have been used to assess the effect of Cx43 on CV. Some studies have shown unaltered CV [5,6], whereas others have reported significantly lower CV [7,8] with reduced Cx43 expression. Studies in mouse models with conditional cardiac knock-out of Cx43 or heterogeneous expression of CX43 have reported an association between decreased Cx43 and arrhythmia generation [9,10]. These and other in vivo studies have suggested that not only the amount of Cx43 content but also its spatial distribution in the tissue is important for arrhythmia generation [9,11,12]. Indeed, the spatial distribution of CX43 has been correlated with the presence of ventricular arrhythmias in patients with chronic heart failure [11]. Also, the CX43 distribution within cardiomyocytes has been related to arrhythmic risk. Seidel et al. [13] reported a significant increase in lateral CX43 in human atrial fibrillation, accompanied by a significant increase in transversal CV, suggesting that CX43 lateralization may also have a proarrhythmic effect.

Age-related changes in Cx43 characteristics have been investigated in animal models, with divergent results depending on the animal species and the age groups compared. A decrease in Cx43 content, expressed as the percentage of Cx43 in cardiac tissue, has been reported in aged or adult mice [14,15] and rats [16] compared to young animals. In addition, an age-related decrease in Cx43 expression levels has been described in mice [15] and rats [17]. Regarding Cx43 lateralization, only Dhein and Hammerath [18] reported an increase in aged compared to young rabbits. In humans, however, there is a lack of studies on the quantitative and spatial (within cardiomyocytes and across the myocardium) changes of CX43 with age and of their effects on cardiac electrophysiology. The paucity of studies, in both animals and humans, is more pronounced from middle age, although the risk of arrhythmias increases significantly compared to young people, probably as a result of the cumulative effect of genetics and/or environment over time [19].

Cardiomyocytes in the left ventricle (LV) are embedded in the extracellular matrix, which provides tissue strength and cell support and allows cell-to-cell contact [20]. Age has been associated with increased extracellular matrix deposition in both animal and human LV [21–24], which alters cardiac electromechanical function and increases susceptibility to arrhythmias [24,25]. The observations that cardiac aging is associated with decreased Cx43 and increased fibrotic content have led to the postulation of a relationship between the two [26,27]. Using aged mice with 100% or 50% Cx43 expression, more pronounced fibrosis was observed in the aged mice with 50% Cx43 expression [28]. However, there is no description of this interaction with age in humans. Alterations in Cx43 expression or fibrosis formation can increase arrhythmia predisposition [1,29], as cardiac impulse propagation requires a fine interplay between electrical coupling, cardiomyocyte excitability, and tissue architecture. Increased fibrosis, which alters impulse propagation due to the different properties of the replacement tissue compared to the normal myocardium [30,31], has been associated with increased arrhythmic risk in human hearts both in the presence of disease [32,33] and in relation to aging [24]. Although mouse models have suggested a possible interplay between reduced Cx43 and fibrosis accumulation in arrhythmogenesis, with both factors being required for the generation of arrhythmias [34,35], the implications that this may have in humans is yet unclear.

The aim of this study is to characterize CX43 remodeling beyond midlife in the human LV, including its relationship with fibrosis, and ultimately to determine the influence that CX43 characteristics have on conduction, repolarization duration, and dispersion and arrhythmicity via computational modeling. We consider age not only from a chronological standpoint, but also from a biological perspective using the age marker lipofuscin [24,36]. Our data indicate that significant CX43 remodeling is not present with age from midlife onwards in human myocardium, but, at the individual level, CX43 amount and heterogeneity combined with fibrosis deposition are major contributors to cardiac arrhythmogenicity.

## Results

### CX43 amount, expression level, lateralization, and spatial heterogeneity present high interindividual variability

**Fig. 1.**
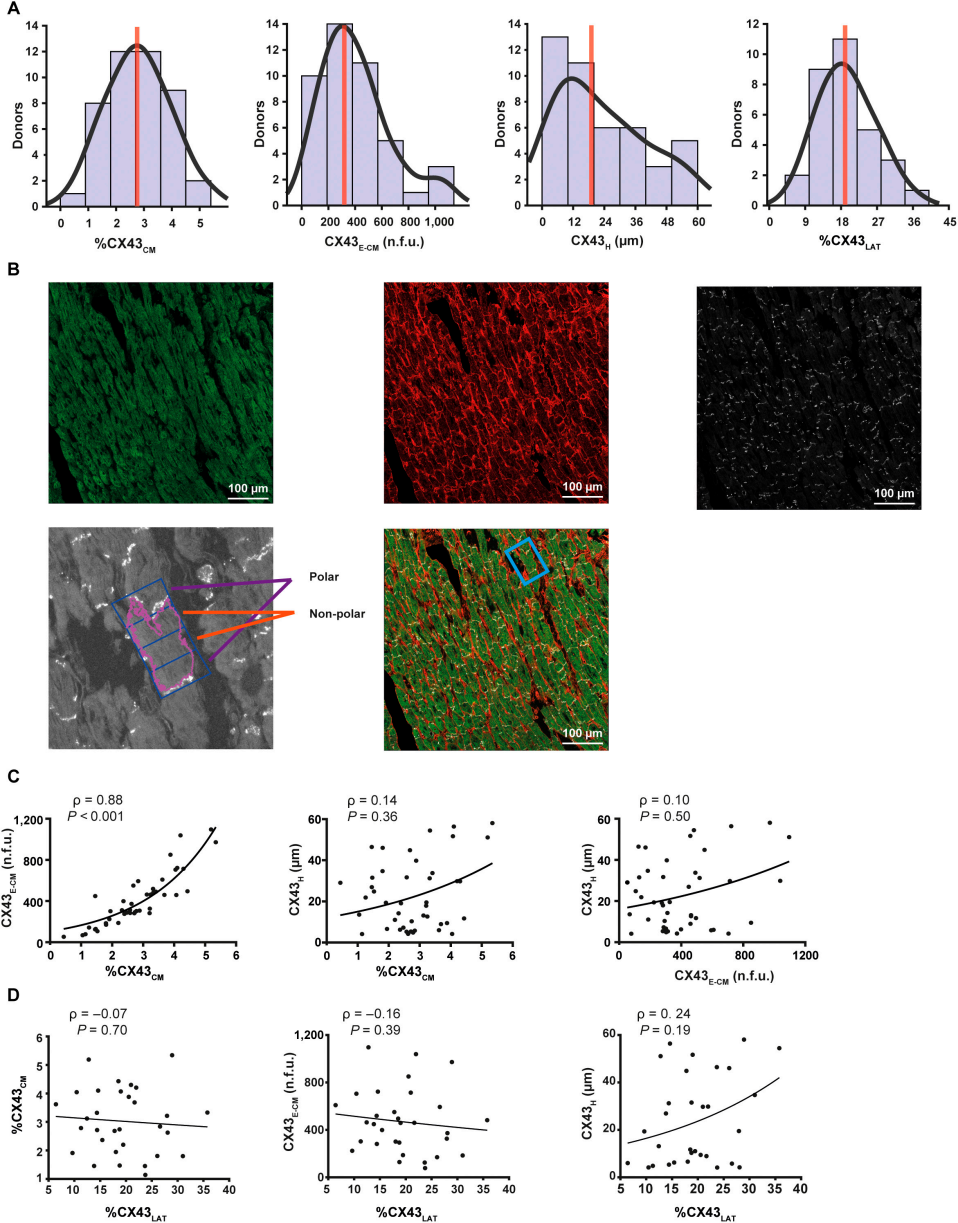
Quantification of CX43 features with respect to the cardiomyocyte area. (A) Distribution of CX43 amount (%CX43_CM_), CX43 expression level (CX43_E-CM_), CX43 heterogeneity (CX43_H_), and CX43 lateralization (%CX43_LAT_) in the population. (B) Top row: fluorescence immunohistochemistry staining of the individual channels of SERCA2, WGA, and CX43 (left to right top row) in an LV tissue section. Bottom row: representative image of a cardiomyocyte identified and delimited in polar and non-polar zones by MARTA software (left) and the location of this cardiomyocyte within the tissue section (right). (C) From left to right: correlation between %CX43_CM_ and CX43_E-CM_ and CX43_H_ and between CX43_E-CM_ and CX43_H_. (D) From left to right: correlation between %CX43_LAT_ and %CX43_CM_, CX43_E-CM_, and CX43_H_. Spearman correlation coefficients (ρ) and *P* values (*P*) are shown.

To investigate CX43 dynamics with age in human myocardium, we first quantified CX43-related features in transmural LV biopsies from 44 patients aged 50 to 84 years and characterized their distribution in the population (Fig. 1A). The amount of CX43 relative to cardiomyocyte area (%CX43_CM_) varied from 0.44% to 5.34% in the study population, with a median of 2.73% and high dispersion, as can be seen in the histogram. The CX43 expression level in cardiomyocytes (CX43_E-CM_) ranged from 52.29 normalized fluorescence units (n.f.u.) to 1,095.87 n.f.u., with a population median of 319.59 n.f.u. CX43 heterogeneity (CX43_H_), which measured the non-uniformity of the CX43 spatial distribution, ranged from 4.12 μm to 58.05 μm in the population, with a median of 19.2 μm. In a reduced number of donors (31 out of 44) whose LV tissue samples were longitudinally oriented, we additionally evaluated the distribution of CX43 within the cardiomyocyte. We measured an index of lateralization that quantifies the average percentage of CX43 in the lateral (non-polar) sides of the cardiomyocytes in each individual (%CX43_LAT_) (Fig. 1B) and provides information on the contribution to transverse conduction. %CX43_LAT_ varied from 6.42% to 35.78%, with a median of 18.78% in the population (Fig. 1A).

The amount of CX43 per cardiomyocyte area, %CX43_CM_, correlated significantly with its expression level CX43_E-CM_ (Spearman correlation coefficient, ρ = 0.88, *P* < 0.001), indicating that it is expressed uniformly across different tissue areas. CX43_H_ did not correlate with either %CX43_CM_ or CX43_E-CM_ (Fig. 1C). %CX43_LAT_ did not correlate with any of the other CX43 features (Fig. 1D).

To understand whether gender influences CX43 remodeling, we compared the expression of CX43 features between males and females in the same age group. There were no statistically significant differences in CX43 dynamics between genders in the age range analyzed (Fig. S1).

### CX43 characteristics are poorly related to chronological and biological age beyond midlife, with increased CX43 heterogeneity being the more distinctive feature in old age

**Fig. 2.**
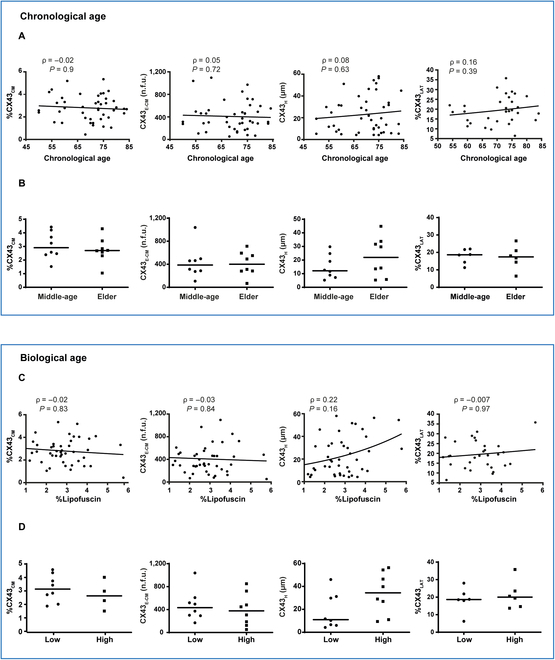
Characterization of CX43 dynamics with chronological and biological age. (A) From left to right: correlation between chronological age and CX43 amount (%CX43_CM_), CX43 expression levels (CX43_E-CM_), CX43 heterogeneity (CX43_H_), and CX43 lateralization (%CX43_LAT_). (B) From left to right: comparison of %CX43_CM_, CX43_E-CM_, CX43_H_, and %CX43_LAT_ in middle-aged versus elder individuals. (C) From left to right: correlation between lipofuscin and %CX43_CM_, CX43_E-CM_, CX43_H_, and %CX43_LAT_. (D) From left to right: comparison of %CX43_CM_, CX43_E-CM_, CX43_H_, and %CX43_LAT_ in individuals with low versus high lipofuscin content. In panels A and C, Spearman correlation coefficients (ρ) and *P* values (*P*) are shown. In panels B and D, black lines represent the median.

We next sought to investigate the relationship between CX43 characteristics (%CX43_CM_, CX43_E-CM_, CX43_H_, and %CX43_LAT_) and chronological age in the human LV tissue. There was no correlation between age and any of the four CX43 characteristics (Fig. 2A). We then compared the lower and upper ends of the age range, corresponding to middle-aged and elderly individuals in our population. A slight non-significant decrease in the %CX43_CM_ was observed in the elder (median [interquartile range (IQR)]: 2.7% [2.45% to 3.13%], *n* = 8) as compared to the middle-aged individuals (median [IQR]: 2.91% [2.43% to 3.95%], *n* = 8). Similarly, CX43_E-CM_ did not change in elderly (median [IQR]: 400.92 [282.55 to 571.68 n.f.u.], *n* = 8) compared to middle-aged subjects (median [IQR]: 386.44 [280.93 to 479.03 n.f.u.], *n* = 8). The median CX43_H_ value increased by 45%, when comparing elder (median [IQR]: 21.96 [9.66 to 32.71 μm], *n* = 8) versus middle-aged individuals (median [IQR]: 12.16 [8.09 to 21.93 μm], *n* = 8), although this difference did not reach statistical significance. No significant differences were either observed for %CX43_LAT_ (median [IQR] in elderly: 17.43% [14.41% to 20.97%], *n* = 6) versus middle-aged (median [IQR]: 18.65% [14.37 to 21.66%], *n* = 6) (Fig. 2B).

To account for age-related effects beyond the mere temporal factor of chronological age, namely, accounting for the effect of genetics and environment, we assessed CX43 characteristics as a function of the aging pigment lipofuscin. Again, no correlation was observed, with only CX43_H_ correlating modestly but not significantly with lipofuscin content (correlation coefficient of 0.22) (Fig. 2C). When comparing the groups representing the individuals with the lowest and highest levels of lipofuscin, we observed a non-significant 16% decrease in the median %CX43_CM_ in the high-lipofuscin group (median [IQR]: 2.54% [1.45% to 3.61%], *n* = 8) versus the low-lipofuscin group (median [IQR]: 3.03% [2.29% to 3.91%], *n* = 8), which was accompanied by a similar non-significant 13% decrease in CX43_E-CM_ in individuals with high lipofuscin (median [IQR]: 377.52 [155.69 to 601.93 n.f.u.], *n* = 8) compared to individuals with low lipofuscin (median [IQR]: 433.75 [296.99 to 563.37 n.f.u.], *n* = 8). The largest differences with biological age were found for CX43_H_, which showed a non-significant 68% increase in the high-lipofuscin group (median [IQR]: 34.41 [18.97 to 50.43 μm], *n* = 8) versus the low-lipofuscin group (median [IQR]: 11.01 [6.30 to 30.51 μm], *n* = 8). Finally, a slight non-significant increase in %CX43_LAT_ was observed in the high-lipofuscin group (median [IQR]: 20% [14.66% to 23.53%], *n* = 6) versus the low-lipofuscin group (median [IQR]: 18.64% [18.04% to 21.86%], *n* = 6) (Fig. 2D).

### CX43 characteristics are not related to fibrosis accumulation beyond midlife

**Fig. 3.**
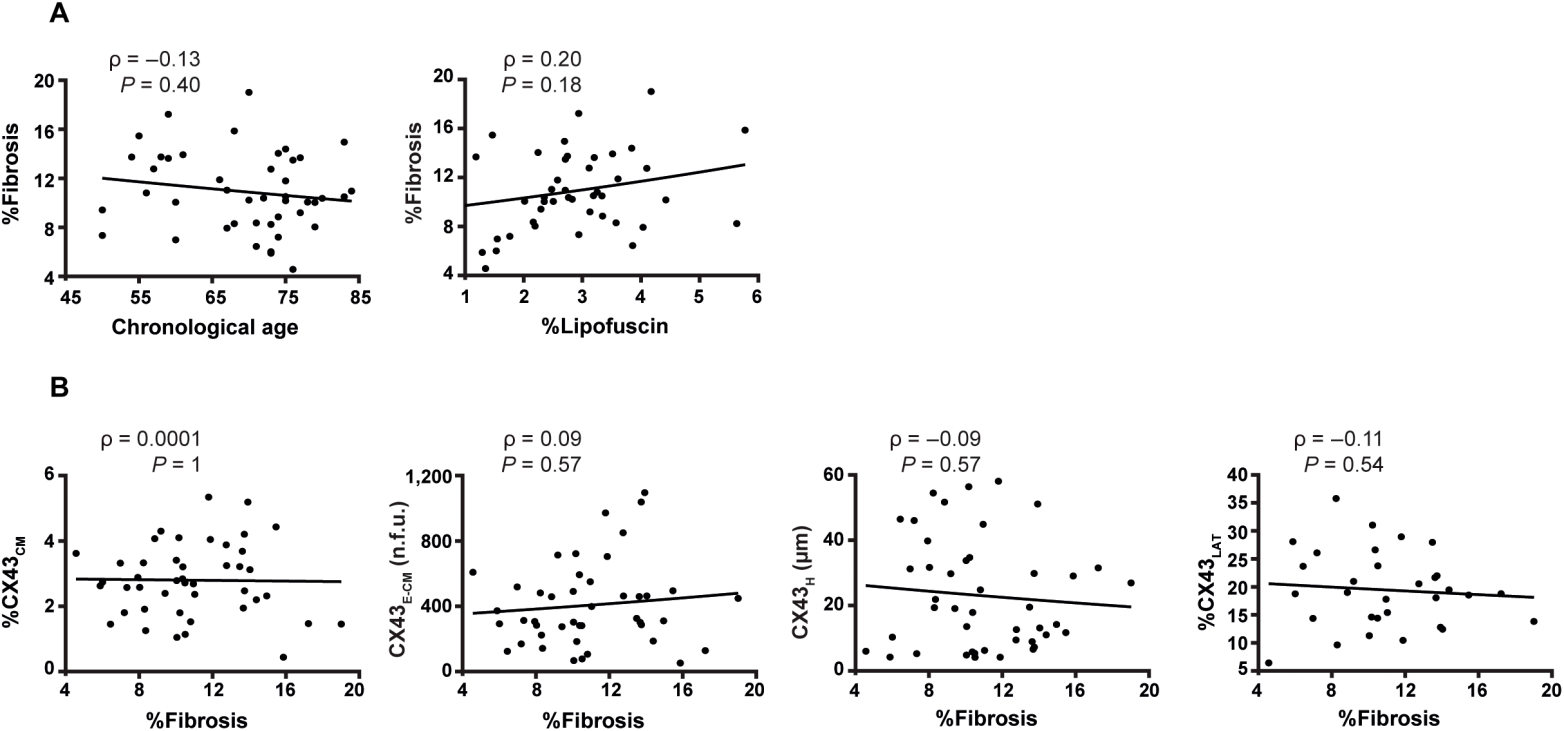
Characterization of CX43 remodeling in relation with fibrosis content. (A) Correlation between fibrosis content and chronological age (left) and lipofuscin amount (right). (B) From left to right: correlation between fibrosis and CX43 amount (%CX43_CM_), CX43 expression levels (CX43_E-CM_), CX43 heterogeneity (CX43_H_), and CX43 lateralization (%CX43_LAT_). Spearman correlation coefficients (ρ) and *P* values (*P*) are shown.

Fibrosis accumulates with age, and it is considered a well-established aging phenotype. In our data, we validated a method to quantify fibrosis content by fluorescence microscopy (Fig. S6) and observed that the percentage of fibrosis beyond midlife did not correlate with chronological age (correlation coefficient of -0.13), whereas it had a modest, non-significant relationship with cardiac biological age (correlation coefficient of 0.2) (Fig. 3A), in agreement with our previous data [22,24]. We also examined the relationship between CX43 characteristics and fibrosis deposition, as previous evidence suggests their interplay with age [28,37]. We did not observe a significant correlation between fibrosis content and any of the CX43-related features: %CX43_CM_, CX43_E-CM_, CX43_H_, or %CX43_LAT_ (Fig. 3B).

In line with previous research evaluating CX43 levels relative to the myocardial tissue area rather than to cardiomyocyte area, we further characterized the relationship of CX43 with age in total tissue (CX43_E-T_). We observed minor changes, with only small, non-significant variations in %CX43_T_ and CX43_E-T_ in elderly versus middle-aged individuals (Fig. S2A). When analyzed by biological age, similar results were observed for %CX43_T_ and CX43_E-T_, with slightly more pronounced decreases in their median values in biologically old versus young individuals (Fig. S2B). The relationship between fibrosis and CX43 amount or expression level was also similar when CX43 quantity was measured by CX43_E-T_ (Fig. S2C).

### CX43 characteristics and fibrosis deposition have marked effects on human ventricular electrical activity

Despite the lack of apparent age-related remodeling of CX43 in our middle-aged to elderly population, we found high interindividual variability in the analyzed CX43 characteristics. We therefore investigated the impact of this variability on cardiac electrophysiology, considering the lower and upper ends of our population in terms of CX43 amount, lateralization, and heterogeneity, either alone or in combination with fibrosis, by assessing the arrhythmic risk associated with these features. We built computational models of human LV electrophysiology and we performed simulations to assess how the amount (defined as %CX43_CM_), lateralization, and heterogeneity of CX43 alone and/or in combination with collagen deposition might influence CV, action potential duration (APD), and arrhythmogenesis in the LV. We did not simulate variations in CX43 expression levels, as CX43_E-CM_ was highly correlated with %CX43_CM_ (Fig. 1C).

Specifically, we used our experimental results to feed 2-dimensional computational models of human LV electrophysiology consisting of epicardial and midmyocardial regions. We defined a control scenario with a longitudinal diffusion coefficient of 0.0013 cm^2^/ms, a transverse-to-longitudinal conductivity ratio of 0.19, and no fibrosis, which was associated with a CV magnitude of 59.71 cm/s, in agreement with experimentally reported values [38]. Changes in CX43 amount and lateralization were implemented in the simulations as variations in the longitudinal diffusion coefficient and transverse-to-longitudinal conductivity ratio, respectively, following approaches previously reported in the literature in both cases [13,39]. In particular, we modeled a 10% to 40% reduction in longitudinal conductivity, mimicking the range of variation in %CX43_CM_ observed in the population. Such a reduction resulted in a 6.31% to 26.85% decrease in the median CV. We also modeled the range of lateralization observed in the population by setting the transverse-to-longitudinal conductivity ratio to 0.23, 0.27, 0.31, and 0.35, without changing the global conductivity (represented by the sum of the longitudinal and transverse conductivities). Increased lateralization led to a 6% to 13.95% increment in CV. When fibrosis was included in the models by randomly assigning some parts of the tissue with fibroblast electrophysiological properties, an inverse correlation between the degree of fibrosis and CV was observed. The lowest level of fibrosis (7%) in our population data caused a 6.16% reduction in CV, and the highest level of fibrosis (19%) caused a 17.79% reduction. We also looked at the effect of combining these 3 factors (reduced longitudinal conductivity, increased transverse-to-longitudinal conductivity ratio, and increased fibrosis) in 4 different levels. The 4 cases resulted in reduced CV (Fig. S3).

**Fig. 4.**
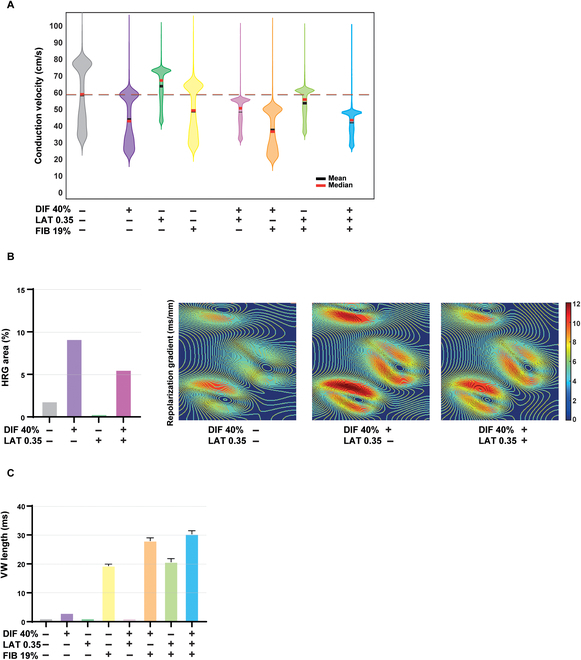
Evaluation of the effect of CX43 remodeling and fibrosis deposition on ventricular electrophysiology and arrhythmic risk. (A) Violin plot of the conduction velocity for the control scenario and for scenarios involving variations in CX43 features (diffusion 40%, DIF 40%; lateralization 0.35, LAT 0.35), fibrosis 19% (FIB 19%), and their combination. (B) Effect of CX43 quantity, lateralization, and the combination of the two on the high repolarization gradient (HRG) area. (C) Effect of CX43 quantity and lateralization, fibrosis, and their combination on the vulnerability window (VW) length. Bar graphs represent mean ± standard deviation.

For the subsequent analysis, we simulated only the extreme cases, that is, the highest reduction in the longitudinal conductivity (40%, henceforth DIF 40%), the highest lateralization (0.35, henceforth LAT 0.35), and the greatest fibrosis percentage (19%, henceforth FIB 19%) individually or combining two or three of them. DIF 40%, FIB 19%, and especially their combination were all associated with a decrease in CV. As LAT 0.35 was associated with a higher CV than the control, the combination of LAT 0.35 with DIF 40%, FIB 19%, or both yielded a higher CV than when LAT 0.35 was not simulated (Fig. 4A).

Regarding APD, we did not observe any changes except for the simulations with fibrosis, which had the shortest APD. This reduction in the mean APD of the tissue, justified by the coupling of cardiomyocytes and fibroblasts and the shorter APD of the fibroblasts, ranged from 33% to 51% for the cases with the lowest and highest percentages of fibrosis, respectively. Similar APD shortening was observed when fibrosis was combined with DIF 40% and/or LAT 0.35 (data not shown).

### Reduced CX43 quantity, increased CX43 heterogeneity, and greater fibrosis deposition increase arrhythmic risk

Next, we evaluated the role of the interindividual variability in CX43 and fibrosis deposition in arrhythmogenesis by evaluating the repolarization gradient and the vulnerability window (VW). For each simulated scenario, we calculated the percentage of tissue with a repolarization gradient greater than an empirically established threshold of 9 ms/mm, which we termed as areas of high repolarization gradient (HRG) following previous studies of the literature [40]. It should be noted that this measure is considered to be a metric of dispersion of repolarization and a surrogate for arrhythmic risk [41]. When we simulated FIB 19%, we observed a decrease in the HRG area compared to control due to the different properties between epicardial and midmyocardial nodes. However, the addition of fibrosis in the same proportion and distribution in a tissue with uniform cell properties would be associated with an increase in the HRG area (Fig. S3A for an epicardial tissue). When we simulated DIF 40% and LAT 0.35, we observed an increase and a decrease in the HRG area, respectively, compared to control while the combination of both led to an intermediate situation (Fig. 4B). As another proarrhythmic risk indicator, we calculated VW, defined as the time interval in which, if a premature ventricular stimulus is delivered, it can lead to a reentrant arrhythmia. DIF 40%, LAT 0.35, and their combination had either little or no effect on VW. FIB 19% was associated with higher VW. The combination of FIB 19% with LAT 0.35 and/or DIF 40% further increased VW (Fig. 4C).

**Fig. 5.**
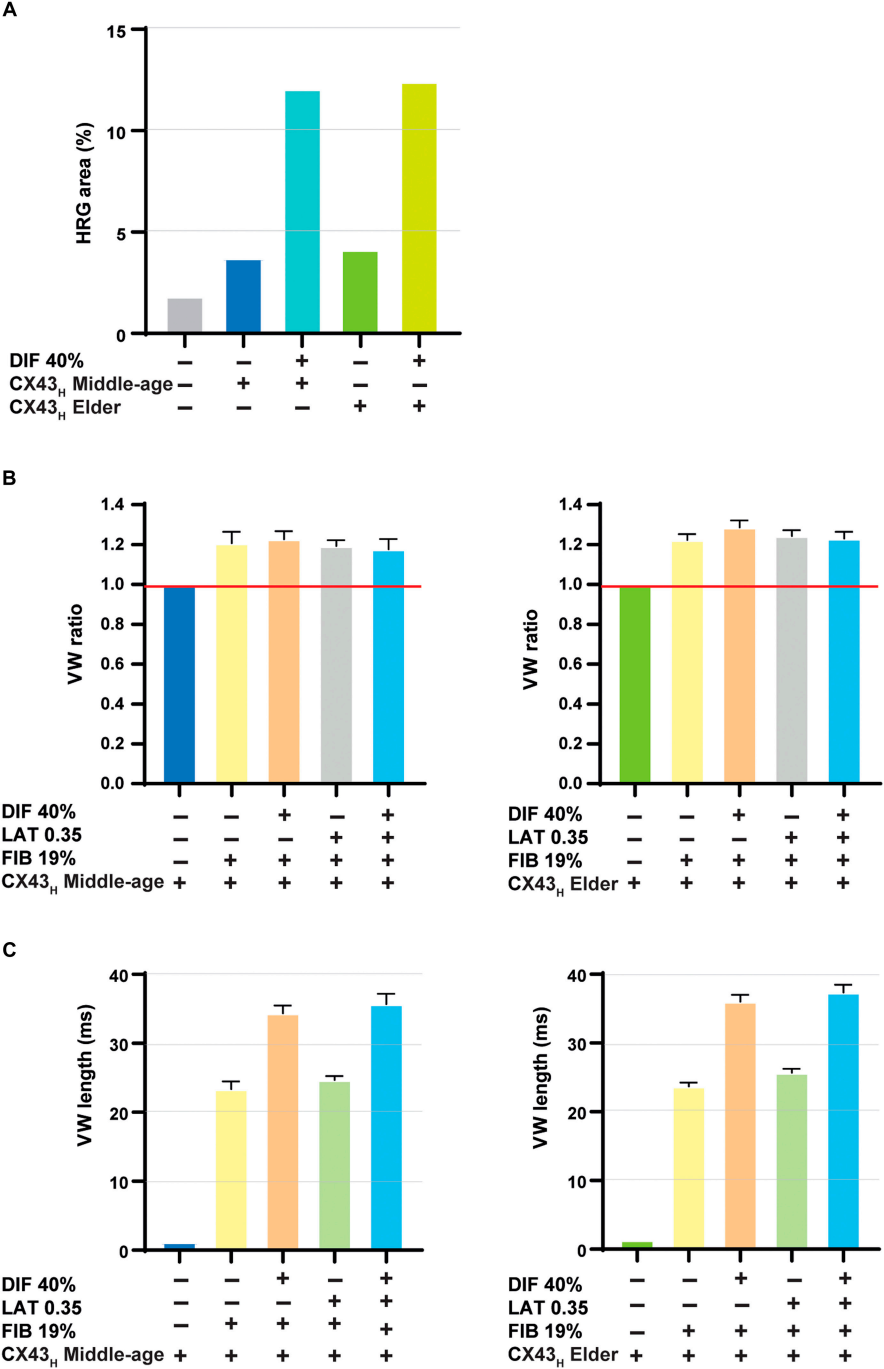
Analysis of the effect of CX43 spatial heterogeneity combined with fibrosis and CX43 quantity and lateralization on repolarization gradient and reentrant arrhythmias. (A) Effect of CX43 heterogeneity (CX43_H_), quantity (%CX43_CM_) (diffusion, DIF 40%), and lateralization (%CX43_LAT_) (LAT 0.35) on HRG area. (B) Effect of CX43 heterogeneity (CX43_H_) in middle-aged (left) and elder (right) individuals together with %CX43_CM_ (diffusion, DIF 40%) and %CX43_LAT_ (LAT 0.35) and fibrosis (FIB 19%) on VW length relative to the same scenarios without heterogeneity. The red line represents the same scenario without heterogeneity. (C) Effect of CX43_H_ in middle-aged (left) and elder (right) individuals together with %CX43_CM_ (diffusion, DIF 40%) and %CX43_LAT_ (LAT 0.35) and fibrosis (FIB 19%), on VW length. Bar graphs represent mean ± standard deviation.

Finally, we performed another set of simulations to represent the spatial distribution of CX43 in the tissue as in our experimental results. We took the tissue samples from 2 individuals whose CX43_H_ was representative of the middle-aged and old groups, and we created corresponding tissue meshes for electrophysiological simulations. We found that CX43 spatial heterogeneity in middle-age, and even more so in old age, was associated with a larger HRG area than in the control (with no heterogeneity). When either heterogeneity distribution was combined with DIF 40%, the HRG area increased remarkably (Fig. 5A). In terms of VW, CX43_H_ combined with FIB 19%, DIF 40%, and/or LAT 0.35 resulted in increased pro-arrhythmicity compared with the cases without heterogeneity. This increase was slightly more pronounced in the heterogeneity associated with the old group than in the middle-aged group (Fig. 5B). The combination of one of the two CX43_H_ with FIB 19% or the 3 factors, i.e., DIF 40%, FIB 19%, and LAT 0.35, led to the greatest effect on VW (Fig. 5C). Spiral waves for one of the simulated cases are shown in Fig. S3b.

## Discussion

### Absence of CX43 remodeling beyond midlife with high interindividual variability

We developed and applied methods to quantify CX43 characteristics in a population of middle-aged to old individuals and we investigated the role of such characteristics on cardiac electrical activity. Our results showed the absence of CX43 remodeling associated with chronological and biological age in the human LV beyond midlife, with high interindividual variability in all age segments. Biological age markers are reported to better describe age-related changes than chronological age in heart tissue [22,24,42]. Consistent with this concept, lipofuscin age pigment seems to underscore changes in tissue remodeling (i.e., CX43_H_ and the fibrosis content) that are just peered or unseen by chronological age, but they are still non-significant. Given the large number of individuals analyzed in our cohort (higher than in many other human studies in cardiac disease) and the high sensitivity of biological age markers, we can conclude that, if present, these changes would be so slight at the population level, which are most likely obscured by the interindividual variability.

The origin of such variability is uncertain. According to our data, it is unlikely to arise from regional LV differences, as all tissue samples corresponded to the same LV region and transmural depth, gender (at least beyond 70 years old), ethnicity (all Caucasian), or degree of fibrosis. Cx43 could also be expressed in other cell types, as it is known in all vascular cell types of various organs such as brain, retina, or kidney [43]. However, as our histological data show, CX43 in human myocardium is mainly localized in the intercalated disks between cardiomyocytes. Furthermore, given that the determination of CX43 quantity relative to total tissue and cardiomyocyte area (with fibrosis values of up to almost 20% in our cohort) was equivalent, we can assume that the amount of CX43 located in non-cardiomyocytes is negligible and has no impact on interindividual variability [43]. At the molecular level, the control of CX43 expression and function is known to imply epigenetic, transcriptional, post-transcriptional (microRNAs), translational, and post-translational mechanisms [44]. Understanding such processes could provide new insights into the basis of the interindividual variability and thus the proarrhythmic potential of CX43. In summary, individual variations in CX43 remodeling from midlife might be better explained, not essentially by age, but by other factors related to the large heterogeneity in the genetic background of the donors and environmental conditions, which would require further investigation.

Comparing our findings with previous research proves difficult. As far as we know, there are no studies characterizing CX43 remodeling with age in the human LV and only limited research is available from animal models. Some animal studies have reported a decrease in Cx43 amount with age [14–16]. Most available studies evaluating Cx43 expression levels only perform qualitative assessments [15,17], studies assessing Cx43 heterogeneity changes with age are scarce [10], and there are no studies on age-related Cx43 lateralization in humans and just a few in animal models, all of them reporting an increase of Cx43 in the lateral membrane of the cardiomyocytes with age [18,45]. Besides, previous research on animal models has compared young animals with either adult or old ones, but there is a paucity of studies comparing adult and old animals despite the age range from midlife onwards being precisely the one expected to be most clinically relevant in terms of cardiac risk.

Regarding the relationship between CX43 characteristics and fibrosis, age is well-known to promote cardiac fibrosis [25] and the amount of fibrosis is reported to inversely correlate the one of CX43 [27]. In agreement with our data [24], we only observed an increase in fibrosis from midlife with biological age; however, it was non-significant. The lack of significance in the current data could rely on the broader specificity of the current fluorescence-based method for components of the extracellular matrix as compared to the collagen-specific second-harmonic-generation microscopy [24]. Each method captures different aspects of cardiac fibrosis even if they report the same trend. Contrary to findings in animal models [27], we did not observe any correlation between CX43 remodeling and fibrosis content, suggesting that, in healthy human ventricular myocardium, no regulatory interplay between the extracellular matrix and CX43 exists from midlife onwards. Whether disease alters this relation in humans and could, thus, be a potential pharmacological target remains to be elucidated. Given the species differences, further studies that investigate this mechanism would require the use of human models like, e.g., cardiac cells derived from human-induced pluripotent stem cells.

Altogether, we provided novel and relevant insights into age-related CX43 and fibrosis dynamics and their interplay in the human LV, which constitutes a major progress over investigations carried out so far, with most studies being conducted in animal models and based on chronological age.

### Role of CX43 characteristics and fibrosis in arrhythmogenesis

Based on the results of our experimental characterizations, we simulated a range of physiological values for CX43 characteristics and fibrosis deposition using mathematical modeling of human LV tissue electrophysiology. The observed reduction in CV in response to a reduction in CX43 quantity is consistent with previous in vivo [7,8] and in silico studies [39]. The effects of larger CX43 lateralization on increased CV also agree with studies using animal models [4,46] and in silico models [13]. The reduced CV associated with increased fibrosis is in agreement with previous studies, which, however, reported greater effects [39], possibly due to the assignment of fibrotic properties to the elements of the simulated tissue.

Our simulations did not lead to an association between CX43 remodeling changes in APD. Increased fibrosis, however, notably reduced APD, in accordance with other in silico studies [47,48]. Thus, the physiological range of variation of human CX43 features has an effect on ventricular electrical conduction but not on repolarization duration, as could be expected.

For arrhythmic risk assessment, we evaluated the repolarization gradient and quantified the percentage of LV tissue with HRG using a threshold of 9 ms/mm. Previous studies have used thresholds ranging from 3 ms/mm [40] to 12.4 ± 3.5 ms/mm in heart failure [49] or 25 ms/mm at the edge of the border zone [50]. Also, we evaluated the length of the VW to assess the probability of a premature ventricular stimulus to induce reentrant arrhythmias. In our simulations, %CX43_LAT_ increased VW length, but it reduced the HRG area. A disparity of evidence can be found in the literature regarding the effects of %Cx43_LAT_. Some authors have reported an increase in CV associated with higher lateralization [4,13,46], whereas others have reported a decrease in CV [51,52]. In our study, CX43_H_ is the CX43 feature whose values change to a larger extent with age. When we simulated spatial heterogeneity in CX43, we found that it contributed to the proarrhythmicity enhanced by high fibrosis content and reduced CX43 quantity. Overall, our simulated data indicate that physiological values of CX43 features and fibrosis can synergistically increment the risk of ventricular arrhythmias in humans.

### Study limitations

The absence of CX43 remodeling from midlife makes it wonder whether extending the cohort size to include individuals in the lower and upper ends of the age range would yield additional information. In particular, the analysis of samples from young individuals could allow us to confirm whether there is an evident remodeling when comparing young and adult or elder individuals, as occurs in animal species. Studies on LV tissues from living donors have the limitation that the donors are not completely healthy (limiting the access to young individuals), but the advantage of being unaffected by post-mortem protein degradation. In this study, biopsies were collected from a region far from the areas irrigated by the obstructed artery in coronary artery disease patients. Besides, our simulations on CX43 heterogeneity were based on representative samples from middle-aged and elderly groups. Further research could take this as a basis and generate in silico models that included CX43 heterogeneity based on the characterizations from a larger number of individuals, which would allow a more accurate deciphering of its potential arrhythmogenic effect.

## Conclusion

We characterized CX43 remodeling with age by assessing its content, expression level, heterogeneity, and lateralization. Although biological age was more strongly correlated with some CX43 characteristics than chronological age, we generally observed non-significant CX43 remodeling with age from midlife, thus ruling out a population-level role of CX43 as a potential arrhythmic modulator in relation to age. Nevertheless, we observed high interindividual variability in CX43 characteristics at all age segments. We integrated our experimental CX43 characterizations, individually and in combination with our observations on fibrosis deposition, into 2-dimensional human LV electrophysiological models. We found that a physiological reduction in the amount of CX43, an increase in its heterogeneity, and a greater fibrosis deposition led to an increased arrhythmic risk.

## Materials and Methods

### Collection and processing of left ventricular tissues

Human transmural LV tissue specimens were collected at Hospital Universitario Miguel Servet (Zaragoza, Spain) from 44 patients older than 18 years old undergoing valve repair or replacement surgery or coronary artery bypass graft. All of them presented preserved systolic function and absence of ventricular hypertrophy or dilated cardiomyopathy. The clinical characteristics of the patients are summarized in Table. The samples were obtained with a 14-G tru-cut biopsy needle during cardiac arrest, immediately after the patient was placed on cardiopulmonary bypass, from an area of the anterior wall of the LV, near the base of the heart, with no evidence of ischemia or any other macroscopic pathology [53]. The specimens were then transferred to 4% paraformaldehyde fixative solution for 1 h at 4 °C and subsequently embedded in paraffin blocks. Tissue sections of 5 μm were mounted on microscope glass slides for ulterior label-free imaging or histochemical procedures.

**Table.** Clinical characteristics of the patients

Gender	Age	Clinical history	LVEF	Ventricular hypertrophy	Dilated cardiomyopathy
Female	70	Aortic valve stenosis	Preserved	No	No
Female	74	Coronary artery disease	Preserved	No	No
Female	75	Coronary artery disease	Preserved	No	No
Female	76	Coronary artery disease	Preserved	No	No
Female	77	Coronary artery disease	Preserved	No	No
Female	79	Aortic valve stenosis	Preserved	No	No
Female	83	Aortic valve stenosis	Preserved	No	No
Female	83	Aortic valve stenosis	Preserved	No	No
Male	50	Coronary artery disease	Preserved	No	No
Male	50	Aortic valve stenosis	Preserved	No	No
Male	54	Coronary artery disease	Preserved	No	No
Male	55	Coronary artery disease	Preserved	No	No
Male	56	Coronary artery disease	Preserved	No	No
Male	57	Coronary artery disease	Preserved	No	No
Male	58	Coronary artery disease	Preserved	No	No
Male	59	Coronary artery disease	Preserved	No	No
Male	59	Coronary artery disease	Preserved	No	No
Male	60	Coronary artery disease and aortic valve stenosis	Preserved	No	No
Male	60	Coronary artery disease	Preserved	No	No
Male	61	Coronary artery disease	Preserved	No	No
Male	66	Coronary artery disease	Preserved	No	No
Male	67	Coronary artery disease and aortic valve stenosis	Preserved	No	No
Male	67	Coronary artery disease	Preserved	No	No
Male	68	Coronary artery disease and aortic valve stenosis	Preserved	No	No
Male	68	Coronary artery disease	Preserved	No	No
Male	70	Coronary artery disease	Preserved	No	No
Male	71	Coronary artery disease and aortic valve stenosis	Preserved	No	No
Male	71	Coronary artery disease and aortic valve stenosis	Preserved	No	No
Male	72	Coronary artery disease	Preserved	No	No
Male	73	Coronary artery disease	Preserved	No	No
Male	73	Coronary artery disease	Preserved	No	No
Male	73	Coronary artery disease	Preserved	No	No
Male	73	Coronary artery disease	Preserved	No	No
Male	74	Coronary artery disease and aortic valve stenosis	Preserved	No	No
Male	74	Aortic valve stenosis	Preserved	No	No
Male	75	Aortic valve stenosis	Preserved	No	No
Male	75	Coronary artery disease	Preserved	No	No
Male	75	Coronary artery disease	Preserved	No	No
Male	76	Aortic valve stenosis	Preserved	No	No
Male	77	Aortic valve stenosis	Preserved	No	No
Male	78	Coronary artery disease	Preserved	No	No
Male	79	Aortic valve stenosis	Preserved	No	No
Male	80	Coronary artery disease	Preserved	No	No
Male	84	Coronary artery disease	Preserved	No	No

Collection and analysis of human LV samples conformed to the principles outlined in the Declaration of Helsinki and were approved by the local ethics committee (CEICA, reference number PI17/0023), with all patients giving written informed consent before surgery and prior to their inclusion in the study. For comparison purposes, 2 age groups were established: the youngest individuals formed the middle-aged group (50 to 59 years old, *n* = 8) and the oldest individuals formed the elder group (77 to 84 years old, *n* = 8). Samples from all individuals were used for correlation analysis (*n* = 44).

### Histochemical procedures

For fluorescence immunohistochemistry, tissue sections were stained following standard protocols. Briefly, samples were deparaffinated by 2 washes with xylol and 4 steps with ethanol 100%–100%–96%–70%. Samples were rehydrated first with tap water followed by distilled water before antigen retrieval with citrate buffer for 30 min. Then, samples were blocked for 1 h with Protein Block Serum-Free (X090930-2, Agilent, USA). Primary antibody incubation was performed at 4 °C overnight, and after 3 washes with 0.5% bovine serum albumin (BSA) in phosphate buffered saline (PBS), secondary antibody incubation was performed for 1 h at room temperature in darkness. Primary antibodies were mouse monoclonal anti-SERCA2 (1:1,000, ab2817, Abcam, UK) and rabbit polyclonal anti-CX43 (1:1,000, ab11370, Abcam, UK). Secondary antibodies were Alexa Fluor 488 goat anti-mouse (1:1,000, A11029, Thermo Fisher, USA) and Alexa Fluor 633 goat anti-rabbit (1:1,000, A21071, Thermo Fisher, USA). The extracellular matrix was stained with Alexa Fluor 555 wheat germ agglutinin (WGA) (1:500, W32464, Thermo Fisher, USA). To reduce variability, a single mix for primary and secondary antibodies and washing buffer was prepared and all sections were stained simultaneously. Images were acquired with a confocal microscope Zeiss LSM 880 (Carl Zeiss, Germany) and the parameter settings for the imaging were kept constant for all the samples.

Regarding histochemistry, picrosirius red was performed automatically in all samples (ST5020 Stainer Integrated Workstation, Leica, Germany) using standard protocols [54].

### Label-free imaging of lipofuscin and tissue autofluorescence

For each individual, a serial section of the one used for the multiple fluorescent immunohistochemistry was used for the label-free imaging of tissue autofluorescence and lipofuscin. The acquisitions were performed with the confocal microscope Zeiss LSM 880 (Carl Zeiss, Germany) and all the parameter settings were kept constant between samples. The wavelength used for excitation and detection of the tissue autofluorescence was 488 and 499 to 553 nm, respectively. For lipofuscin, excitation was at 633 nm and emission was at 650 to 735 nm.

### Image analysis

The procedure followed for the analysis of CX43, fibrosis, and lipofuscin characteristics was the following. Images were carefully checked before being included in the analysis and large vessels surrounded by perivascular fibrosis were removed. Two independent observers performed all image processing and quantification analysis. Each observer established the thresholds for the generation of binary masks, from which computations were performed automatically to compute CX43, SERCA2, WGA, tissue autofluorescence, and lipofuscin characteristics. All the results reported in the manuscript correspond to the average results from the 2 observers. The images of the florescence immunohistochemistry of all the donors are presented in Fig. S5.

#### Quantification of the total amount of CX43 and the percentage of lateralized CX43

Original CZI images were handled with ZEN Blue 2.5 software (Carl Zeiss, Germany). The maximum intensity projections were generated using all planes of the SERCA2, WGA, and CX43 z-stacks. The resulting images were exported as 8-bit raw-TIFF individual images. These were processed for automatic brightness scaling using ImageJ [55] and for removal of isolated image segments (fluorescence artifacts or areas of clear perivascular fibrosis, among others). Next, images were analyzed with the semiautomated open software MARTA [56] to quantify the amount and degree of lateralization of CX43. Briefly, the software MARTA used the separate channels of SERCA2, CX43, and WGA of each individual as TIFF images. Binary masks for CX43 were generated by applying erosion and dilation operations and by establishing a threshold in the CX43 histogram to separate the CX43 signal from the background [56]. Analogously, binary masks for SERCA2 and WGA were generated from the automatically adjusted, gray-level SERCA2 and WGA images by applying a binarization filter.

The amount of CX43 was estimated using 2 different approaches. In the first approach, we quantified the percentage of CX43 with respect to the area of the cardiomyocytes, according to the SERCA2 mask as:

%CX43_CM_ = number of positive CX43 pixels × 100/number of positive SERCA2 pixels

In the second approach, we quantified the percentage of CX43 with respect to the entire left ventricular tissue, which was determined from the SERCA2 and WGA masks, with pixels being positive in both masks counted only once. The percentage of CX43 was calculated as:

%CX43_T_ = number of positive CX43 pixels × 100/(number of positive SERCA2 and WGA pixels*)

* Pixels being positive in both masks counted only once

For quantification of CX43 lateralization, the software MARTA delimited the cardiomyocytes using the SERCA2 and WGA signals [56]. Next, each identified cardiomyocyte was divided into 4 rectangles and MARTA quantified the amount of CX43 in the 2 middle areas of the cardiomyocyte (i.e., the lateral sides) with respect to the total amount of CX43 in the cardiomyocyte (Fig. 1B). MARTA performed this analysis automatically for every cardiomyocyte detected in the tissue section. For each individual, a percentage of CX43 lateralization (%CX43_LAT_) was computed as the average value of the measures in all detected cardiomyocytes. Based on previous studies [3,4,18] and considering that the angle of sectioning can affect the analysis of CX43 lateralization, the inclusion criteria were even more restrictive. Specifically, %CX43_LAT_ was estimated only in tissue samples with primary longitudinally oriented cardiomyocytes (31 out of 44). In addition, an inclusion criterion was applied for %CX43_LAT_ calculation in each tissue sample: only cardiomyocytes confirmed by the observer to be longitudinally oriented were accepted. The set of 31 samples was divided in fifths for the comparison of age groups, with 6 donors in each of the chronological age-based groups, middle-age (54 to 60 years old) and elder (76 to 84 years old), and in each of the biological age-based groups (lowest and highest lipofuscin content).

#### Quantification of CX43 expression levels

CX43 expression level was quantified in the 16-bit raw-TIFF CX43 images exported from Zen Blue 2.5 using in-house MATLAB code but only in the positive pixels of the CX43 binary masks generated by the software MARTA obtained in the previous section. As above, 2 approaches were used to measure CX43 expression level: relative to the cardiomyocyte area and to the total tissue area (including interstitial tissue). In the first approach, the CX43 expression level was calculated as:

CX43_E-CM_ = sum of intensities of positive CX43 pixels/number of positive SERCA2 pixels

In the second approach, the CX43 expression level was made relative to the sum of positive SERCA2 and WGA pixels to account for the whole LV tissue:

CX43_E-T_ = sum of intensities of positive CX43 pixels/(number of positive SERCA2 and WGA pixels*)

* Pixels being positive in both masks counted only once

In both cases, the quantification of the expression levels followed an approach similar to that used in other works of the literature [57].

#### Quantification of CX43 heterogeneity

CX43 heterogeneity (CX43_H_) was quantified using in-house MATLAB code implementing the method proposed by Boulaksil [11]. The method departed from the CX43 binary mask generated by the software MARTA and calculated, for each positive CX43 pixel, the shortest Euclidean distance to the next positive CX43 pixel. The standard deviation of all shortest distances of all pixels was used as a measure of CX43_H_.

#### Quantification of fibrosis percentage

Fibrosis quantification from fluorescence immunohistochemistry images was performed using WGA staining [58], as in previous studies evaluating cardiac fibrosis [59]. Image analysis was based on our previous approximation for collagen quantification using non-linear optical microscopy [24]. Briefly, taking the above-described SERCA2 and WGA binary masks as an input, an in-house MATLAB code was used to compute the number of positive WGA pixels not positive in the SERCA2 mask to obtain the WGA signal corresponding with fibrosis (Fig. S6). Then, we applied the following formula to calculate the percentage of fibrosis:

%Fibrosis = number of pixels only positive to WGA × 100/(number of positive SERCA2 and WGA pixels*)

* Pixels being positive in both masks counted only once

Fibrosis content from picrosirius red staining was quantified using an RGB-based method previously developed in our group [22]. Although the staining was automatic, we observed high intersample variability, which made subsequent image analysis challenging (Figs. S7 and S8A). Therefore, we selected 20 samples with the typical picrosirius red staining, i.e., muscle fibers and cytoplasm in yellow and collagen in red (Fig. S7), and the correlation between the 2 methods remarkably increased (Fig. S8B). Both WGA and picrosirius red gave comparable results, and the use of WGA to assess cardiac fibrosis was validated in our study.

#### Quantification of lipofuscin content

The quantification of lipofuscin content was based on a method previously published [24]. Briefly, original CZI images from all donors were handled with ZEN Blue 2.5 software. Both lipofuscin and tissue autofluorescence signals were exported as raw 16-bit TIFF images and were processed with ImageJ. For tissue autofluorescence, images were adjusted to remove the 0.2% of the highest and lowest values of the histogram. Image adjustment for lipofuscin quantification was determined empirically after revising all the samples of the study, and finally only the range 0 to 3,500 of the histogram was used for image analysis. From the obtained gray-scale images, a threshold was established, and binary masks were generated. Lipofuscin content was calculated using in-house MATLAB software as:

%Lipofuscin = number of positive lipofuscin pixels/number of positive tissue autofluorescence pixels

### In silico modeling and simulation of ventricular electrical activity for varying CX43 characteristics and fibrosis percentage

To assess the impact of CX43 features and the amount of fibrosis on cardiac electrical activity, we fed 2-dimensional computational tissue models of human ventricular electrophysiology with our experimental characterizations. We built a 3 × 3 cm^2^ mesh in which all the mesh nodes were assigned with the electrophysiological properties of epicardial cells except for 3 islands composed of nodes with the electrophysiological properties of midmyocardial cells. The O’Hara–Rudy model was used to describe the human ventricular action potential for the 2 types of cells [60]. We defined a control scenario in which the longitudinal diffusion coefficient was set to 0.0013 cm^2^/ms [39] and the transverse-to-longitudinal conductivity ratio was set to 0.19, with this ratio corresponding to the median value of CX43 lateralization in our population.

To model variations in the content of CX43 (%CX43_CM_), we varied the longitudinal diffusion coefficient. We assigned the value 0.0013 cm^2^/ms to the median of the middle-aged group and we performed simulations with a 10%, 20%, 30%, and 40% reduction in the longitudinal conductivity of the tissue based on our experimental data. This was based on the fact that the elder had, in median, 10% lower %CX43_CM_ compared to the median of the middle-aged group, the high-lipofuscin individuals had, in median, a 20% reduction in %CX43_CM_ in comparison with the low-lipofuscin individuals, the high-lipofuscin individuals had 30% lower %CX43_CM_ than the middle-aged group, and the case of 40% decrease corresponded to the lowest %CX43_CM_ in the population compared to the median of the middle-aged group. To model variations in the degree of CX43 lateralization (%CX43_LAT_), we varied the transverse-to-longitudinal conductivity ratio. The median of %CX43_LAT_ in the population, 19%, was set as the control value. We performed simulations with ratios of 0.23, 0.27, 0.31, and 0.35, as these values cover the range from the median to the highest value of the population. To model variations in the percentages of fibrosis, we assigned some of the tissue elements with electrophysiological properties of fibroblasts using a uniform random distribution. The action potential of the fibroblasts was represented by the MacCannell model of mammalian ventricular fibroblasts [61]. We simulated 7%, 11%, 15%, and 19% fibrosis based on our experimental data. Seven percent corresponds to the median of the low-lipofuscin individuals, 11% corresponds to the median of the high-lipofuscin individual, 15% is an intermediate value, and 19% is the maximum fibrosis percentage in the population. The longitudinal conductivity between fibroblasts or between myocytes and fibroblasts was set to be 3 times lower than the conductivity between cardiomyocytes.

To evaluate the impact of variations in CX43 characteristics and fibrosis deposition on CV and APD, we applied a train of stimuli in a region at the left side of the tissue and the middle of the mesh with a size of 120 × 20 nodes. The stimuli had an amplitude of twice the diastolic threshold, had a duration of 2 ms, and were delivered at a cycle length of 1,000 ms. For each of the simulated scenarios, we evaluated the CV magnitude and the APD. CV was determined using a previously reported method [62]. This method was based on fitting a polynomial surface to the activation data and calculated the local velocity vectors from the gradient of the fitted surface. The APD was calculated by measuring the elapsed time between the activation, defined as the time occurrence of the maximum derivative of the action potential upstroke, and the time for 90% repolarization.

### In silico assessment of arrhythmic risk for varying CX43 characteristics and fibrosis percentage

Next, we aimed to assess the role that different CX43 characteristics and fibrosis percentages play on arrhythmic risk. For that purpose, we evaluated the repolarization gradient and the VW for the highest changes in diffusion (DIF 40%), lateralization (LAT 0.35), and fibrosis (FIB 19%) and the combinations of two or three of these factors. From the simulations described in the previous paragraph, the repolarization gradient was estimated, for each pixel, using methods previously described in the literature in which a radius of one pixel was used in the computations [40,41,49]. By setting a threshold of 9 ms/mm on the repolarization gradient, we calculated the area of the tissue with values above that threshold, which we denoted by HRG.

Additionally, we evaluated the length of the VW as a measure of the likelihood for induction of reentrant arrhythmias. We generated 5 × 5 cm^2^ meshes by rescaling the 3 × 3 cm^2^ meshes described above and we investigated the generation of spiral waves after application of an S1–S2 cross-stimulation protocol [63]. In this protocol, we delivered a sequence of periodic S1 stimuli followed by an extra stimulus S2 to assess pro-arrhythmicity due to the interaction of the S2 stimulus wave front with the S1 stimulus wave tail. The S1 stimuli were applied at the left side of the tissue at a time tS1 = 50 ms, with duration td = 2 ms, period tT = 1 s, and amplitude A equal to twice the diastolic threshold. The S2 stimulus was applied on a patch region at the top left corner of the tissue with the same values of td and A as for the S1 stimulus. The S2 stimuli time was varied to compute the VW, defined as the time interval during which the S1–S2 interaction leads to the generation of a sustained spiral wave.

Further arrhythmic risk assessment was performed after including CX43 heterogeneity in the models. Two meshes with CX43 heterogeneity were obtained as follows. We selected the tissue sample of an individual whose measure of CX43_H_ was similar to the median of the middle-aged group and, similarly, we took the tissue sample of an individual from the old group. Based on the CX43 intensities of the sample, we constructed a histogram, and we divided it into 20 bins. The intermediate bin was associated with the median of the histogram and was assigned the longitudinal diffusion coefficient set in control (0.0013 cm^2^/ms). The 20 different values of longitudinal diffusion were assigned randomly to the nodes of the mesh according to the distribution given by the histogram. The sum of the longitudinal diffusion coefficients of all nodes was kept constant between the 3 meshes: the one without heterogeneity in which the longitudinal diffusion coefficient was constant, and the two with heterogeneity representative of the middle-aged and old groups. Regarding VW, we calculated its length and the increment in VW length after including CX43 heterogeneity with respect to the corresponding scenario without heterogeneity. Specifically, the increment was calculated by dividing the VW length of the scenario with heterogeneity by the same scenario without heterogeneity (VW ratio).

Simulations were performed using an in-house cardiac electrophysiology solver, ELECTRA, implementing the finite element method and meshless methods [63,64] for the solution of the cardiac monodomain model [64,65]. In this work, the dual-adaptive explicit integration algorithm [65] was used to efficiently solve the monodomain model.

### Statistical analysis

Spearman correlation analysis was used to test the strength and direction of association between 2 variables. The Mann–Whitney test was used to assess differences between 2 independent groups. The significance threshold was established by setting a *P* value = 0.05 for both Spearman correlation and Mann–Whitney test. Unless otherwise indicated, data are presented as median [IQR].

## Data Availability

All data reported in this paper will be shared by the corresponding author upon request. Any additional information required to reanalyze the data reported in this paper is available from the corresponding author upon request.

## Supplementary Materials

Supplementary 1 Fig. S1. Comparison of CX43 features between male and female donors of the same age range. Fig. S2. Comparison of the analysis of CX43 remodeling with respect to the cardiomyocyte area or the whole LV tissue. Fig. S3. Evaluation of conduction velocity for all simulated scenarios involving reduction in the longitudinal diffusion coefficient, increase in transverse-to-longitudinal diffusion ratio, increase in the content of fibrosis, and the combination of these 3 factors. Fig. S4. Repolarization gradient on epicardial meshes. Fig. S5. Images of the fluorescence immunohistochemistry of all the donors. Fig. S6. Methodology used for fibrosis quantification. Fig. S7. Images of picrosirius red histochemistry used to validate WGA-based fibrosis quantification method. Fig. S8. Validation of the WGA-based method of fibrosis quantification with picrosirius red staining.
