# Supplementary material for: Interindividual Age-Independent Differences in Human CX43 Impact Ventricular Arrhythmic Risk
Source: Research (Wash D C). 2023 Nov 15;6:0254. doi: 10.34133/research.0254 (PMC10650968; doi:10.34133/research.0254)
Supplement: Supplementary 1 — Fig. S1. Comparison of CX43 features between male and female donors of the same age range. Fig. S2. Comparison of the analysis of CX43 remodeling with respect to the cardiomyocyte area or the whole LV tissue. Fig. S3. Evaluation of conduction velocity for all simulated scenarios involving reduction in the longitudinal diffusion coefficient, increase in transverse-to-longitudinal diffusion ratio, increase in the content of fibrosis, and the combination of these 3 factors. Fig. S4. Repolarization gradient on epicardial meshes. Fig. S5. Images of the fluorescence immunohistochemistry of all the donors. Fig. S6. Methodology used for fibrosis quantification. Fig. S7. Images of picrosirius red histochemistry used to validate WGA-based fibrosis quantification method. Fig. S8. Validation of the WGA-based method of fibrosis quantification with picrosirius red staining. [file research.0254.f1.zip › SM Figure caption.docx]

Supplementary Materials

**Supplemental Figures**

**Figure S1. Comparison of CX43 features between male and female donors of the same age range. a)** CX43 amount (%CX43_CM_), **b)** CX43 expression level (CX43_E-CM_), **c)** CX43 heterogeneity (CX43_H_) and **d)** CX43 lateralization (%CX43_LAT_). Bar graphs represent median ± interquartile range.

**Figure S2. Comparison of the analysis of CX43 remodeling with respect to the cardiomyocyte area or the whole LV tissue. a)** From left to right: representation of CX43 amount (%CX43) and expression level (CX43_E_) versus chronological age, and quantification of %CX43 and CX43_E_ in middle-aged versus elder individuals. **b)** From left to right: representation of %CX43 and CX43_E_ versus lipofuscin content and quantification of %CX43 and CX43_E_ in low versus high lipofuscin individuals. **c)** Representation of fibrosis accumulation and %CX43 (left) and CX43_E_ (right). Spearman correlation coefficients (Rho) and p-values (p) are shown. Black dots and squares correspond to values relative to the cardiomyocyte area, while blue dots and squares are relative to the whole LV tissue.

**Figure S3. Evaluation of conduction velocity for all simulated scenarios involving reduction in the longitudinal diffusion coefficient, increase in transverse-to-longitudinal diffusion ration, increase in the content of fibrosis and the combination of these three factors.** ‘%Diffusion decrease (DIF)’ stands for an decrease in the longitudinal diffusion coefficient of 10%, 20%, 30% and 40%, respectively, associated to physiological changing amounts of CX43 (%CX43_CM_). ‘Lateralization ratio (LAT)’ stands for an increase in the transverse-to-longitudinal diffusion ratio to 0.23, 0.27, 0.31 and 0.35, respectively, related to physiological variations in the degree of CX43 lateralization (%CX43_LAT_). ‘%Fibrosis (FIB)’ stands for addition of fibrosis by 9%, 11%, 15% and 19%, respectively, representing values found in our cohort. ‘D10/L0.23/F7%’ stands for 10% decrease in DIF, LAT of 0.23 and 7% of FIB, and analogously for the other combinations.

**Figure S4. Repolarization gradient on epicardial meshes. a)** High Repolarization Gradient (HRG) area in a mesh with only epicardial nodes without (left column) or with (right column) 19% fibrosis. **b)** Images presenting the spiral waves generated for one of the simulated scenarios coded for voltage (V) values.

**Figure S5. Images of the fluorescence immunohistochemistry of all the donors.** Each row displays, from left to right, the individual channels (SERCA2, WGA and CX43) and the merge for each donor.

**Figure S6. Methodology used for fibrosis quantification. a)** Top row: Images of fluorescence immunohistochemistry of SERCA2 (left) and WGA (right). Middle row: SERCA2 (left) and WGA (middle) computed masks and the resulting computed mask of fibrosis content (right) derived from the subtraction of SERCA2 signal to the WGA signal. Bottom row: picrosirius red staining (left) and the corresponding tissue and fibrosis computed mask (right) used for RGB-based quantification.

**Figure S7. Images of picrosirius red histochemistry used to validate WGA-based fibrosis quantification method.** Tissue serial sections of the ones used for fluorescence immunohistochemistry stained with picrosirius red. Given the variability in the resulting picrosirius signal, images sharing the standard yellow and red signal (black box) are selected as a separate subset for further correlation analysis.

**Figure S8. Validation of WGA-based method of fibrosis quantification with picrosirius red staining. a)** Correlation between fibrosis quantification using the WGA and picrosirius red-based methods using all the stained tissues. **b)** Correlation between fibrosis quantification using the WGA and picrosirius red-based methods using the subset of 20 selected images with homogeneous picrosirius red signal. Spearman correlation coefficients (Rho) and p-values (p) are shown.
