# Supplementary figures and images for: Interindividual Age-Independent Differences in Human CX43 Impact Ventricular Arrhythmic Risk
Source: Research (Wash D C). 2023 Nov 15;6:0254. doi: 10.34133/research.0254 (PMC10650968; doi:10.34133/research.0254)

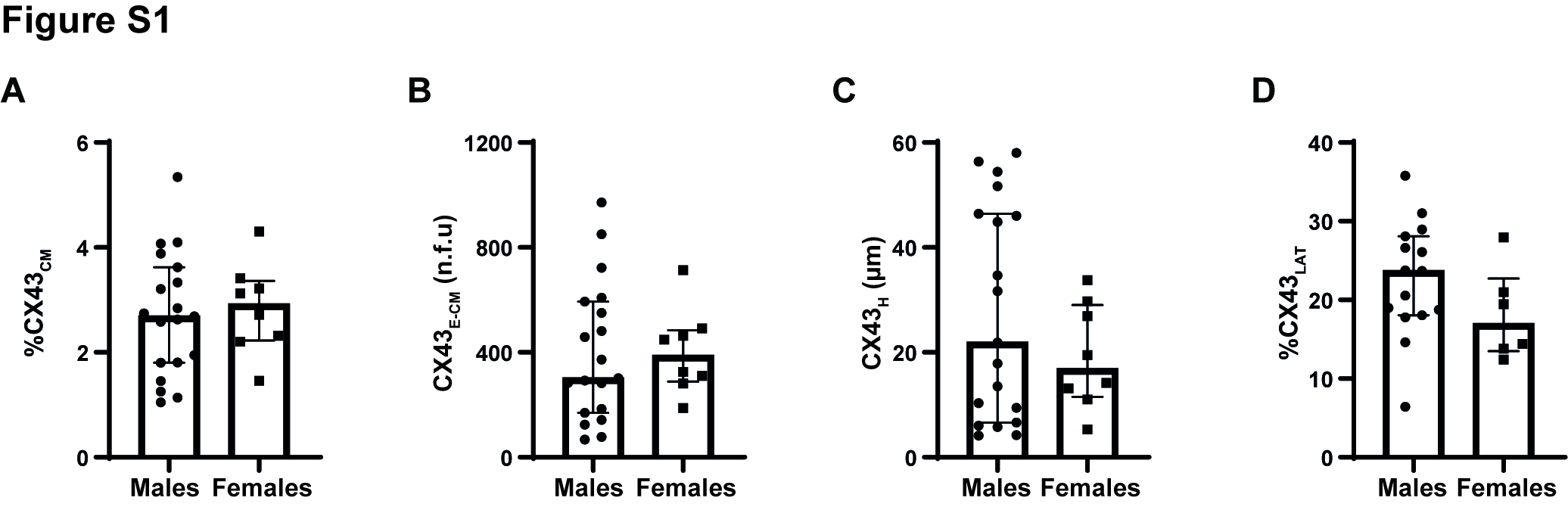

Supplement: Supplementary 1 — Fig. S1. Comparison of CX43 features between male and female donors of the same age range. Fig. S2. Comparison of the analysis of CX43 remodeling with respect to the cardiomyocyte area or the whole LV tissue. Fig. S3. Evaluation of conduction velocity for all simulated scenarios involving reduction in the longitudinal diffusion coefficient, increase in transverse-to-longitudinal diffusion ratio, increase in the content of fibrosis, and the combination of these 3 factors. Fig. S4. Repolarization gradient on epicardial meshes. Fig. S5. Images of the fluorescence immunohistochemistry of all the donors. Fig. S6. Methodology used for fibrosis quantification. Fig. S7. Images of picrosirius red histochemistry used to validate WGA-based fibrosis quantification method. Fig. S8. Validation of the WGA-based method of fibrosis quantification with picrosirius red staining. [file research.0254.f1.zip › Figure S1.tif]

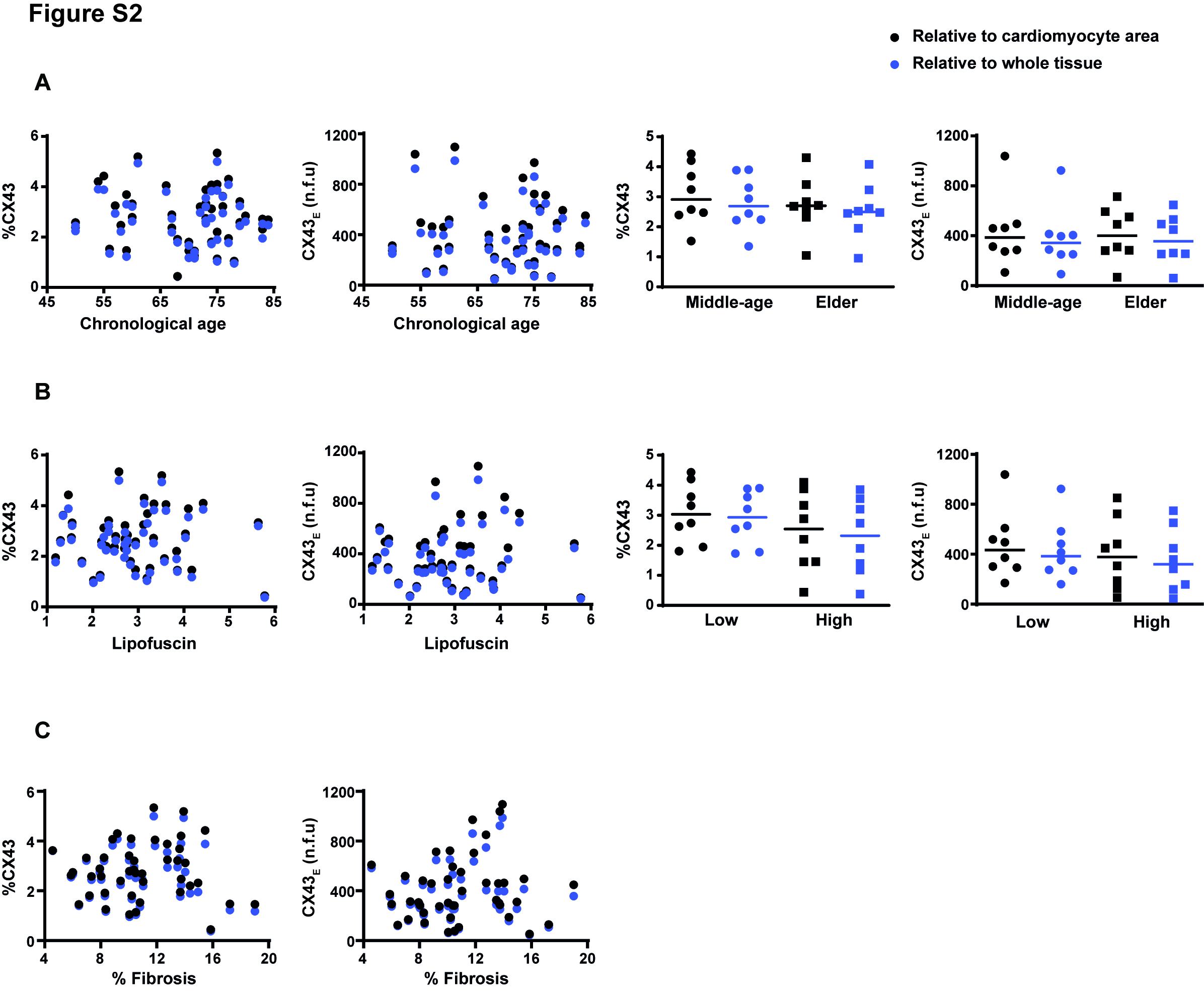

Supplement: Supplementary 1 — Fig. S1. Comparison of CX43 features between male and female donors of the same age range. Fig. S2. Comparison of the analysis of CX43 remodeling with respect to the cardiomyocyte area or the whole LV tissue. Fig. S3. Evaluation of conduction velocity for all simulated scenarios involving reduction in the longitudinal diffusion coefficient, increase in transverse-to-longitudinal diffusion ratio, increase in the content of fibrosis, and the combination of these 3 factors. Fig. S4. Repolarization gradient on epicardial meshes. Fig. S5. Images of the fluorescence immunohistochemistry of all the donors. Fig. S6. Methodology used for fibrosis quantification. Fig. S7. Images of picrosirius red histochemistry used to validate WGA-based fibrosis quantification method. Fig. S8. Validation of the WGA-based method of fibrosis quantification with picrosirius red staining. [file research.0254.f1.zip › Figure S2.tif]

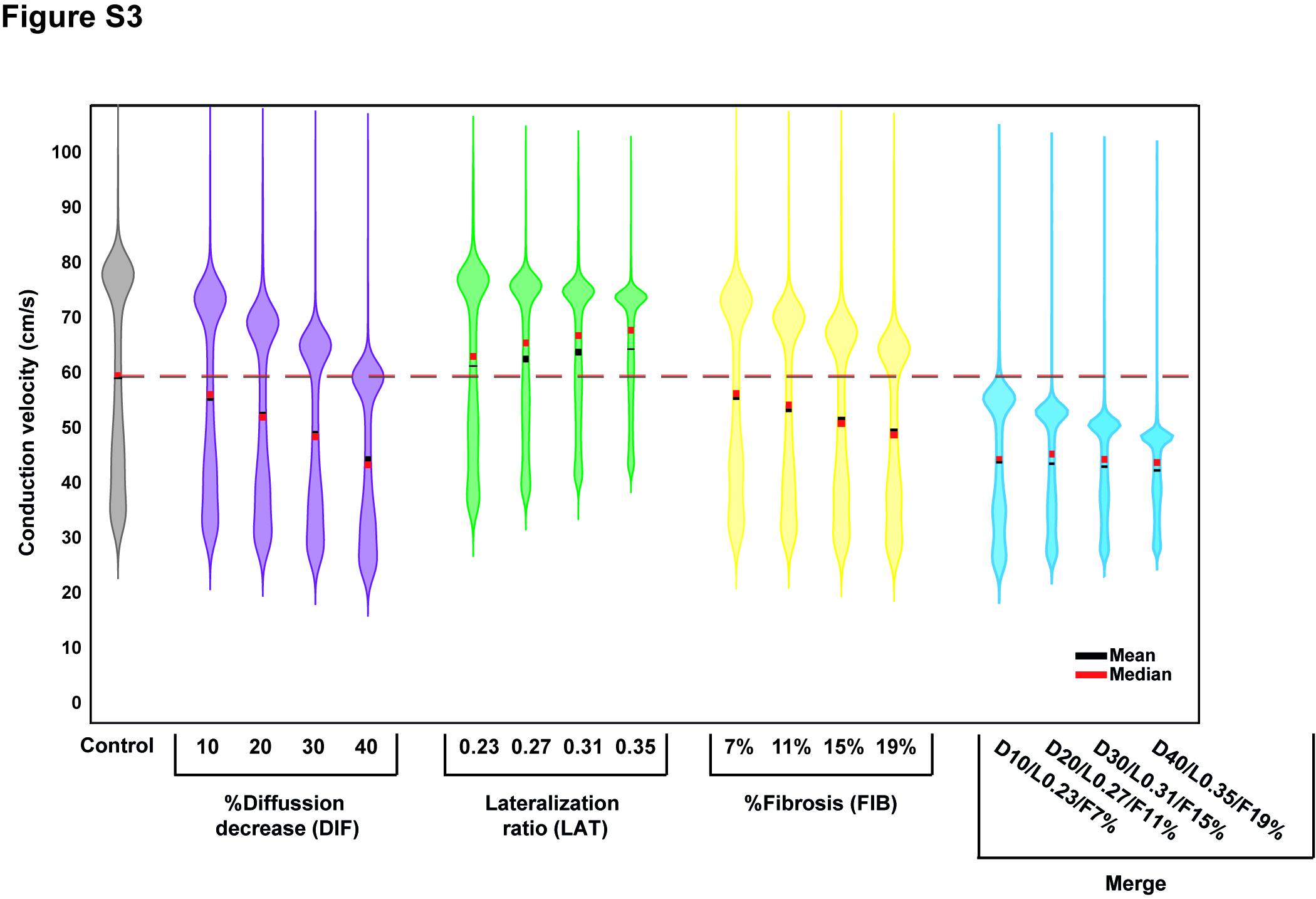

Supplement: Supplementary 1 — Fig. S1. Comparison of CX43 features between male and female donors of the same age range. Fig. S2. Comparison of the analysis of CX43 remodeling with respect to the cardiomyocyte area or the whole LV tissue. Fig. S3. Evaluation of conduction velocity for all simulated scenarios involving reduction in the longitudinal diffusion coefficient, increase in transverse-to-longitudinal diffusion ratio, increase in the content of fibrosis, and the combination of these 3 factors. Fig. S4. Repolarization gradient on epicardial meshes. Fig. S5. Images of the fluorescence immunohistochemistry of all the donors. Fig. S6. Methodology used for fibrosis quantification. Fig. S7. Images of picrosirius red histochemistry used to validate WGA-based fibrosis quantification method. Fig. S8. Validation of the WGA-based method of fibrosis quantification with picrosirius red staining. [file research.0254.f1.zip › Figure S3.tif]

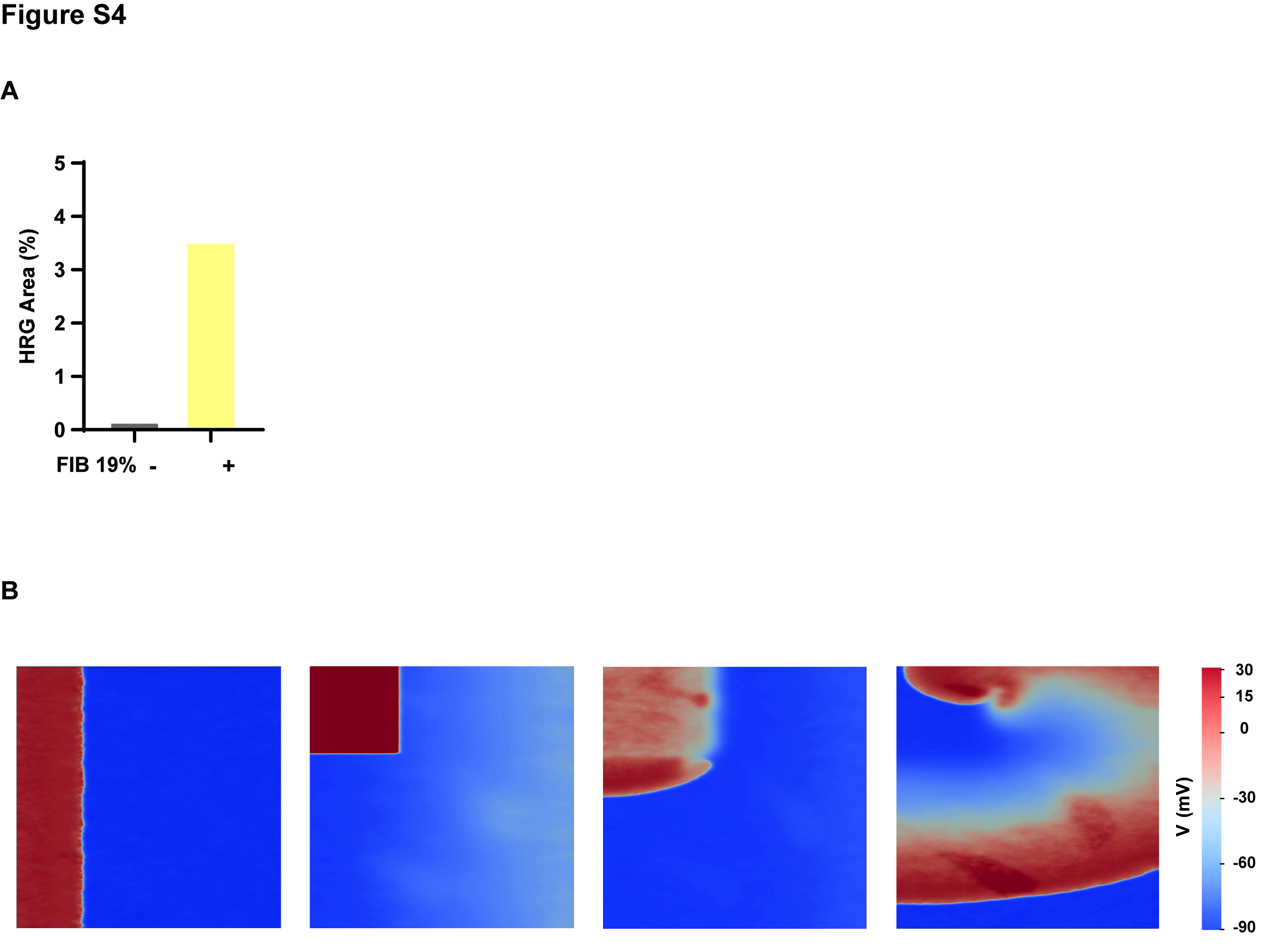

Supplement: Supplementary 1 — Fig. S1. Comparison of CX43 features between male and female donors of the same age range. Fig. S2. Comparison of the analysis of CX43 remodeling with respect to the cardiomyocyte area or the whole LV tissue. Fig. S3. Evaluation of conduction velocity for all simulated scenarios involving reduction in the longitudinal diffusion coefficient, increase in transverse-to-longitudinal diffusion ratio, increase in the content of fibrosis, and the combination of these 3 factors. Fig. S4. Repolarization gradient on epicardial meshes. Fig. S5. Images of the fluorescence immunohistochemistry of all the donors. Fig. S6. Methodology used for fibrosis quantification. Fig. S7. Images of picrosirius red histochemistry used to validate WGA-based fibrosis quantification method. Fig. S8. Validation of the WGA-based method of fibrosis quantification with picrosirius red staining. [file research.0254.f1.zip › Figure S4.tif]

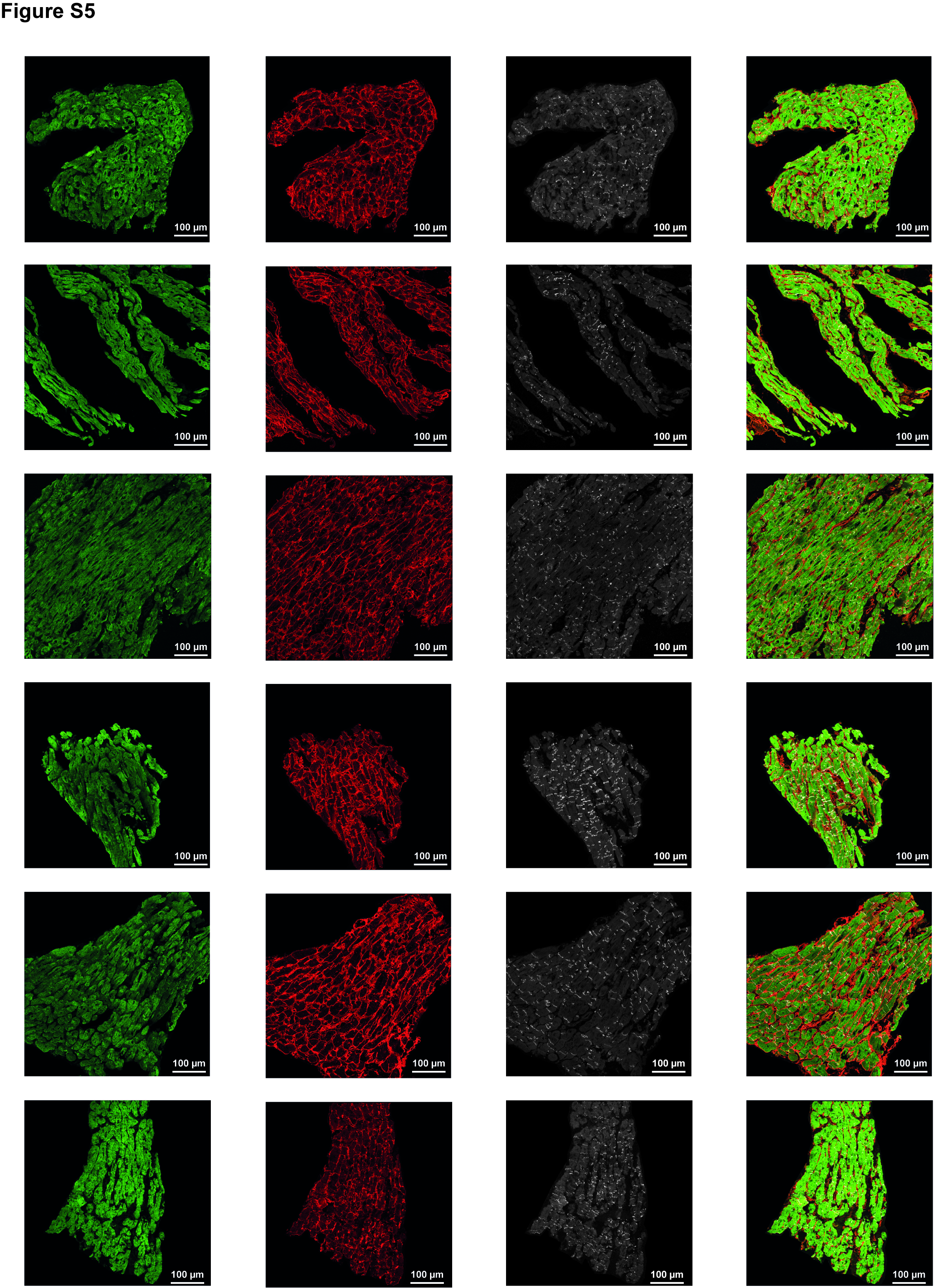

Supplement: Supplementary 1 — Fig. S1. Comparison of CX43 features between male and female donors of the same age range. Fig. S2. Comparison of the analysis of CX43 remodeling with respect to the cardiomyocyte area or the whole LV tissue. Fig. S3. Evaluation of conduction velocity for all simulated scenarios involving reduction in the longitudinal diffusion coefficient, increase in transverse-to-longitudinal diffusion ratio, increase in the content of fibrosis, and the combination of these 3 factors. Fig. S4. Repolarization gradient on epicardial meshes. Fig. S5. Images of the fluorescence immunohistochemistry of all the donors. Fig. S6. Methodology used for fibrosis quantification. Fig. S7. Images of picrosirius red histochemistry used to validate WGA-based fibrosis quantification method. Fig. S8. Validation of the WGA-based method of fibrosis quantification with picrosirius red staining. [file research.0254.f1.zip › Figure S5_1.tif]

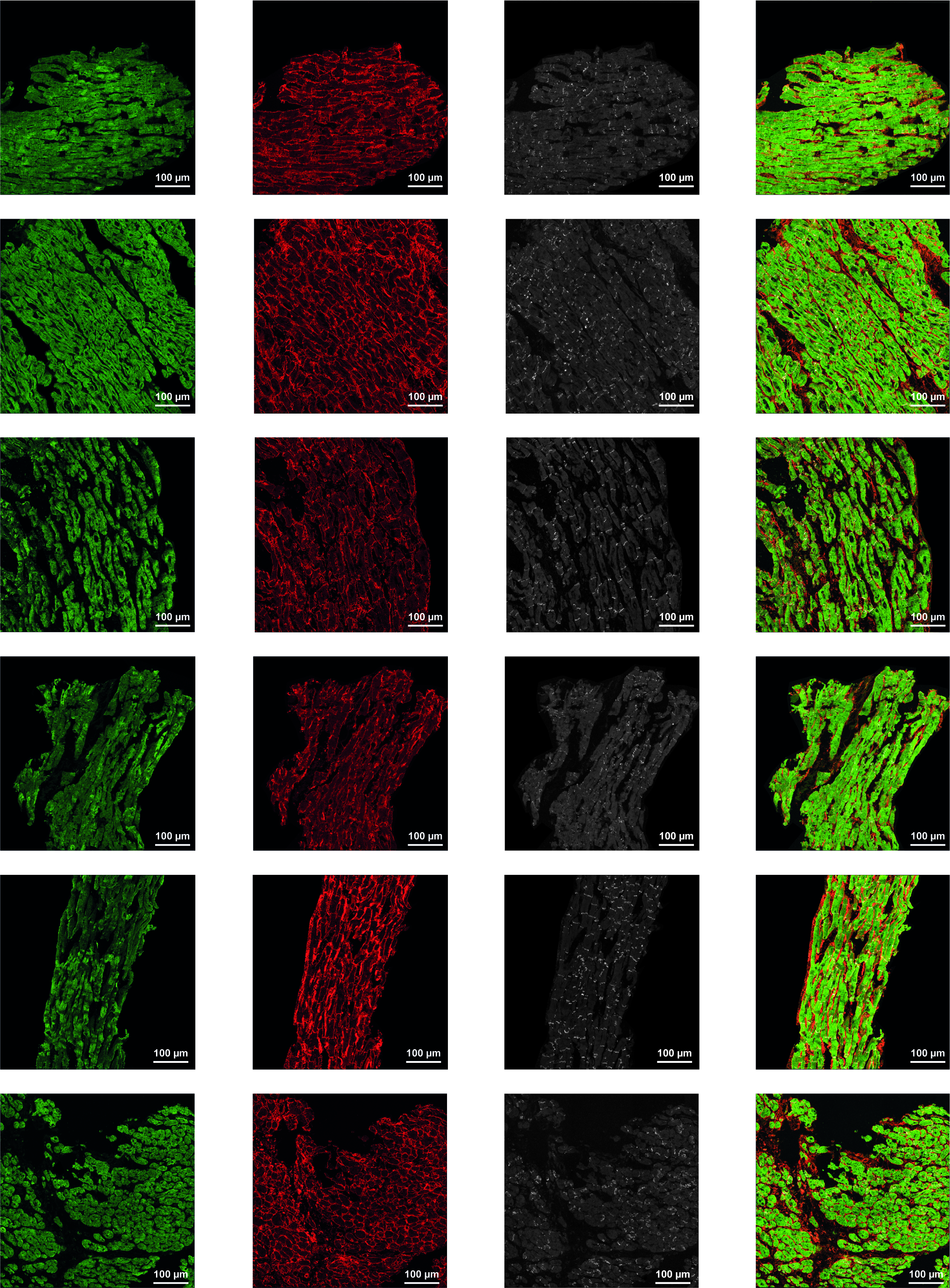

Supplement: Supplementary 1 — Fig. S1. Comparison of CX43 features between male and female donors of the same age range. Fig. S2. Comparison of the analysis of CX43 remodeling with respect to the cardiomyocyte area or the whole LV tissue. Fig. S3. Evaluation of conduction velocity for all simulated scenarios involving reduction in the longitudinal diffusion coefficient, increase in transverse-to-longitudinal diffusion ratio, increase in the content of fibrosis, and the combination of these 3 factors. Fig. S4. Repolarization gradient on epicardial meshes. Fig. S5. Images of the fluorescence immunohistochemistry of all the donors. Fig. S6. Methodology used for fibrosis quantification. Fig. S7. Images of picrosirius red histochemistry used to validate WGA-based fibrosis quantification method. Fig. S8. Validation of the WGA-based method of fibrosis quantification with picrosirius red staining. [file research.0254.f1.zip › Figure S5_2.tif]

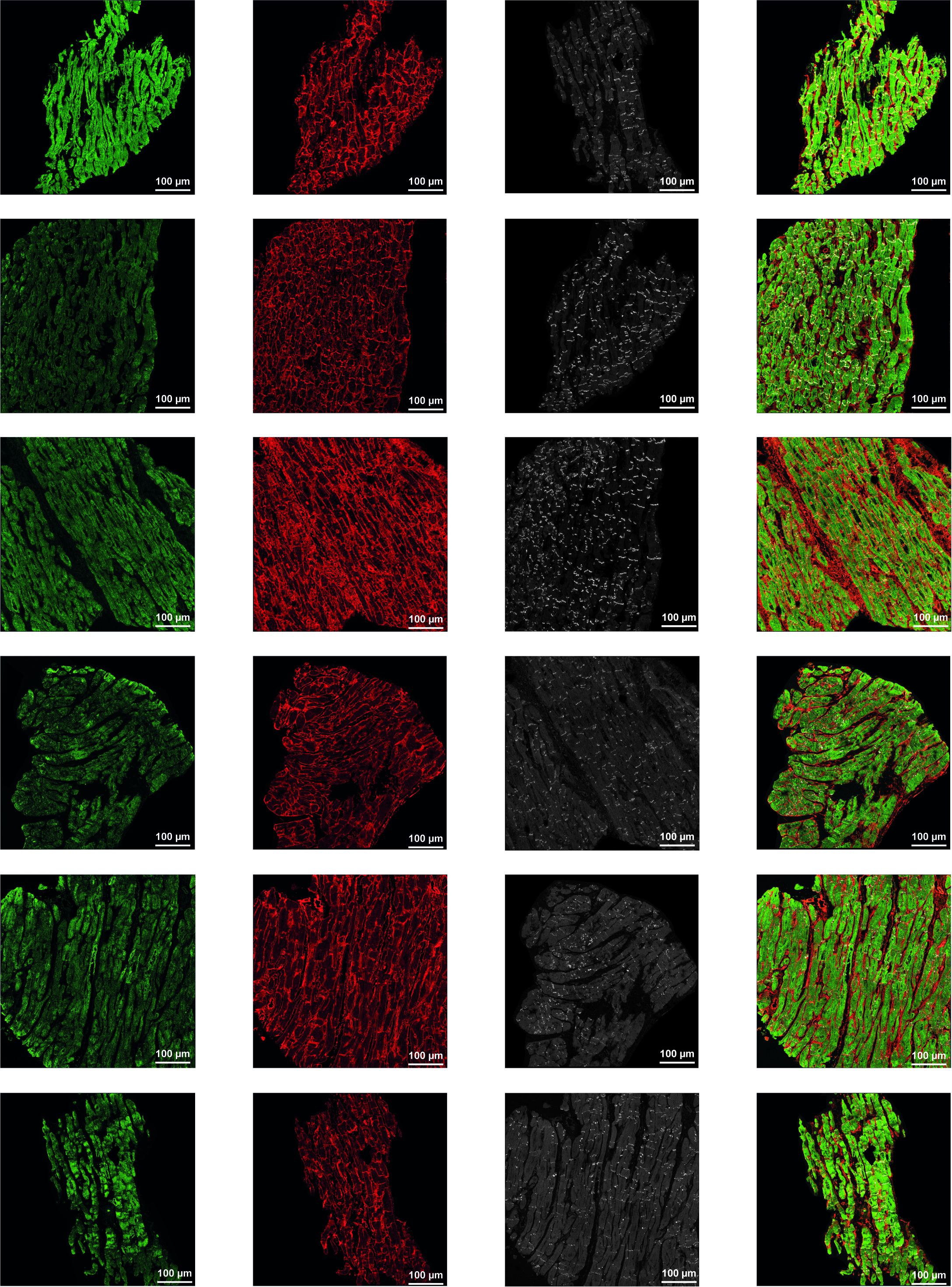

Supplement: Supplementary 1 — Fig. S1. Comparison of CX43 features between male and female donors of the same age range. Fig. S2. Comparison of the analysis of CX43 remodeling with respect to the cardiomyocyte area or the whole LV tissue. Fig. S3. Evaluation of conduction velocity for all simulated scenarios involving reduction in the longitudinal diffusion coefficient, increase in transverse-to-longitudinal diffusion ratio, increase in the content of fibrosis, and the combination of these 3 factors. Fig. S4. Repolarization gradient on epicardial meshes. Fig. S5. Images of the fluorescence immunohistochemistry of all the donors. Fig. S6. Methodology used for fibrosis quantification. Fig. S7. Images of picrosirius red histochemistry used to validate WGA-based fibrosis quantification method. Fig. S8. Validation of the WGA-based method of fibrosis quantification with picrosirius red staining. [file research.0254.f1.zip › Figure S5_3.tif]

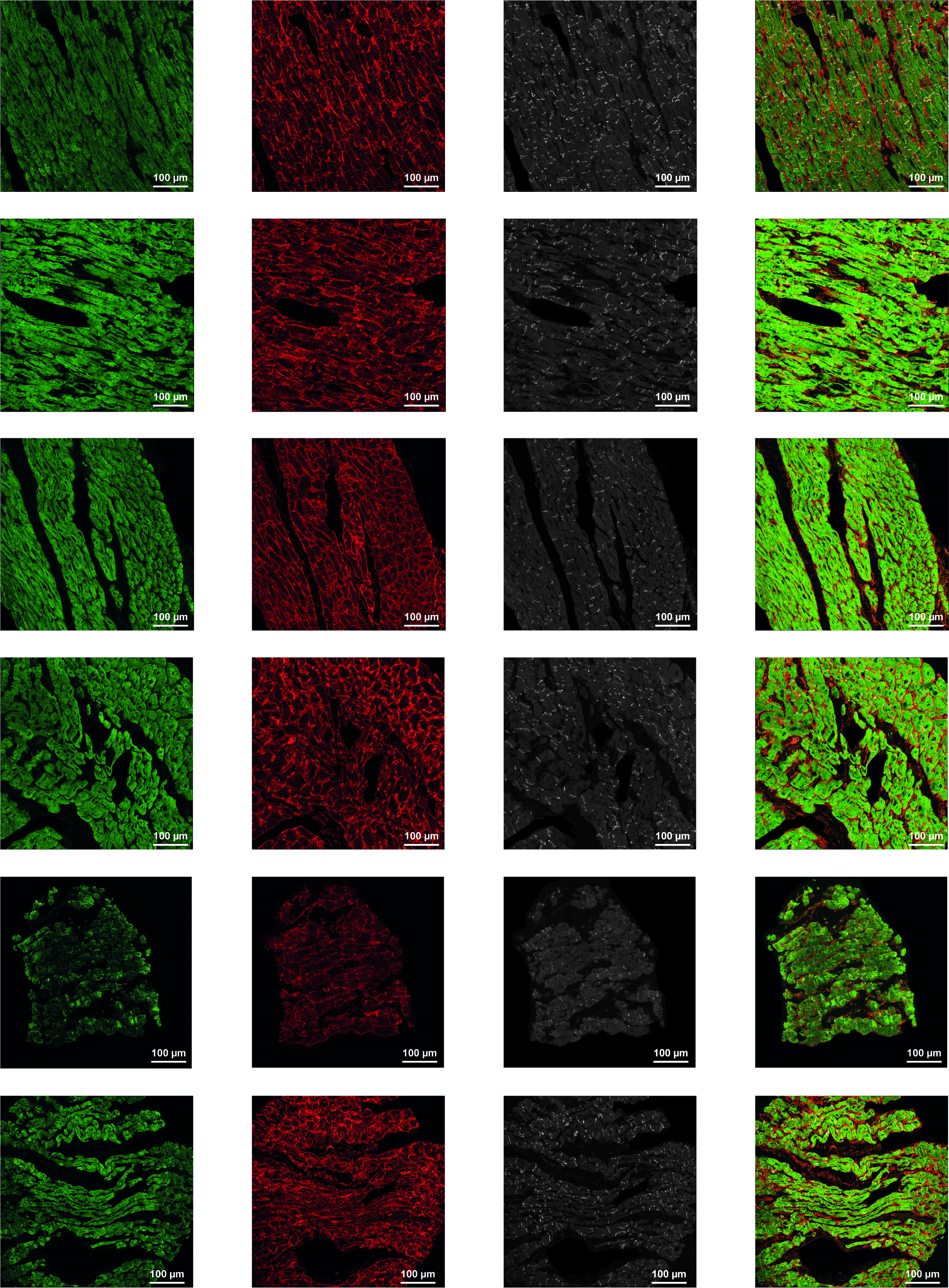

Supplement: Supplementary 1 — Fig. S1. Comparison of CX43 features between male and female donors of the same age range. Fig. S2. Comparison of the analysis of CX43 remodeling with respect to the cardiomyocyte area or the whole LV tissue. Fig. S3. Evaluation of conduction velocity for all simulated scenarios involving reduction in the longitudinal diffusion coefficient, increase in transverse-to-longitudinal diffusion ratio, increase in the content of fibrosis, and the combination of these 3 factors. Fig. S4. Repolarization gradient on epicardial meshes. Fig. S5. Images of the fluorescence immunohistochemistry of all the donors. Fig. S6. Methodology used for fibrosis quantification. Fig. S7. Images of picrosirius red histochemistry used to validate WGA-based fibrosis quantification method. Fig. S8. Validation of the WGA-based method of fibrosis quantification with picrosirius red staining. [file research.0254.f1.zip › Figure S5_4.tif]

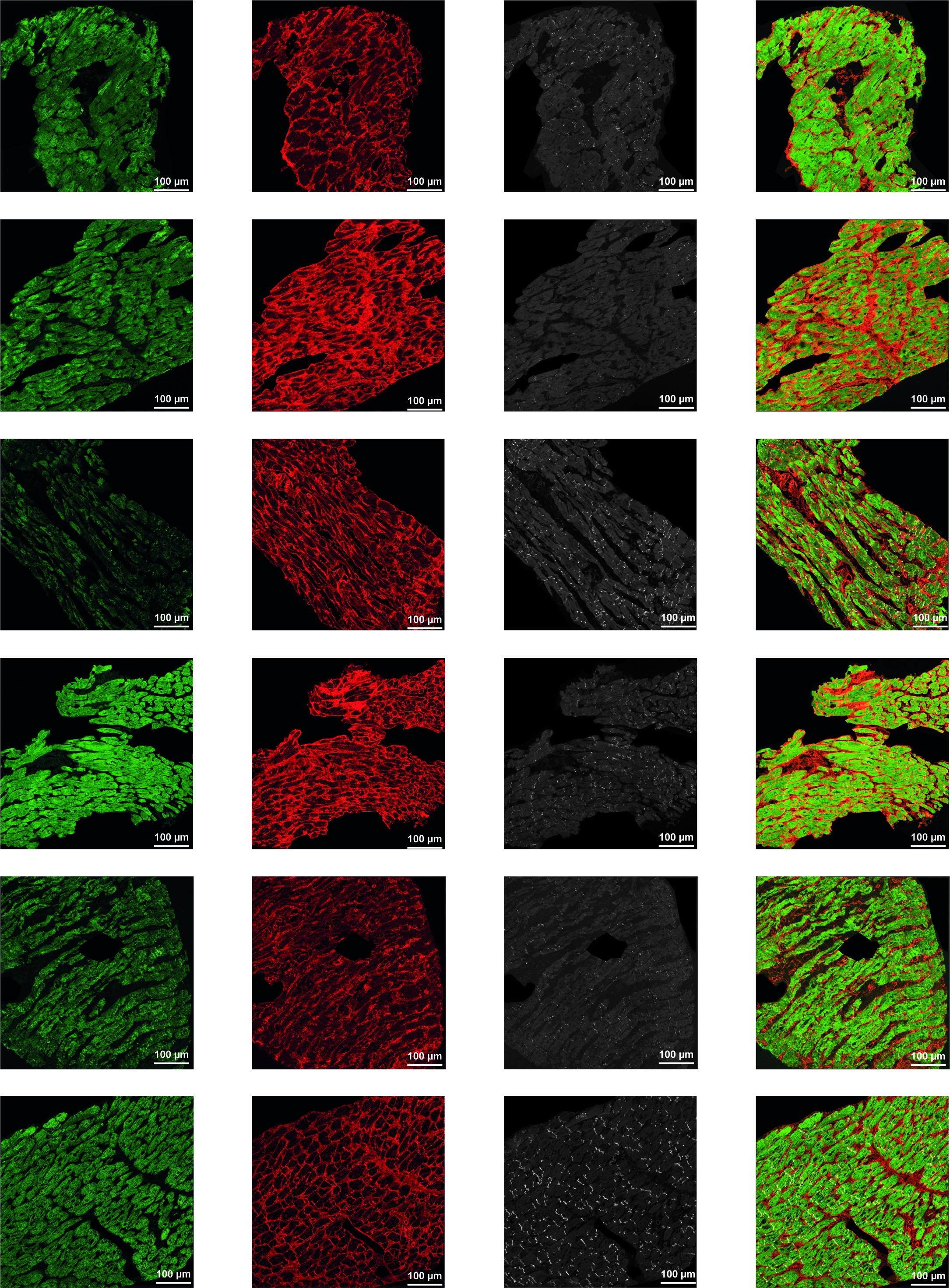

Supplement: Supplementary 1 — Fig. S1. Comparison of CX43 features between male and female donors of the same age range. Fig. S2. Comparison of the analysis of CX43 remodeling with respect to the cardiomyocyte area or the whole LV tissue. Fig. S3. Evaluation of conduction velocity for all simulated scenarios involving reduction in the longitudinal diffusion coefficient, increase in transverse-to-longitudinal diffusion ratio, increase in the content of fibrosis, and the combination of these 3 factors. Fig. S4. Repolarization gradient on epicardial meshes. Fig. S5. Images of the fluorescence immunohistochemistry of all the donors. Fig. S6. Methodology used for fibrosis quantification. Fig. S7. Images of picrosirius red histochemistry used to validate WGA-based fibrosis quantification method. Fig. S8. Validation of the WGA-based method of fibrosis quantification with picrosirius red staining. [file research.0254.f1.zip › Figure S5_5.tif]

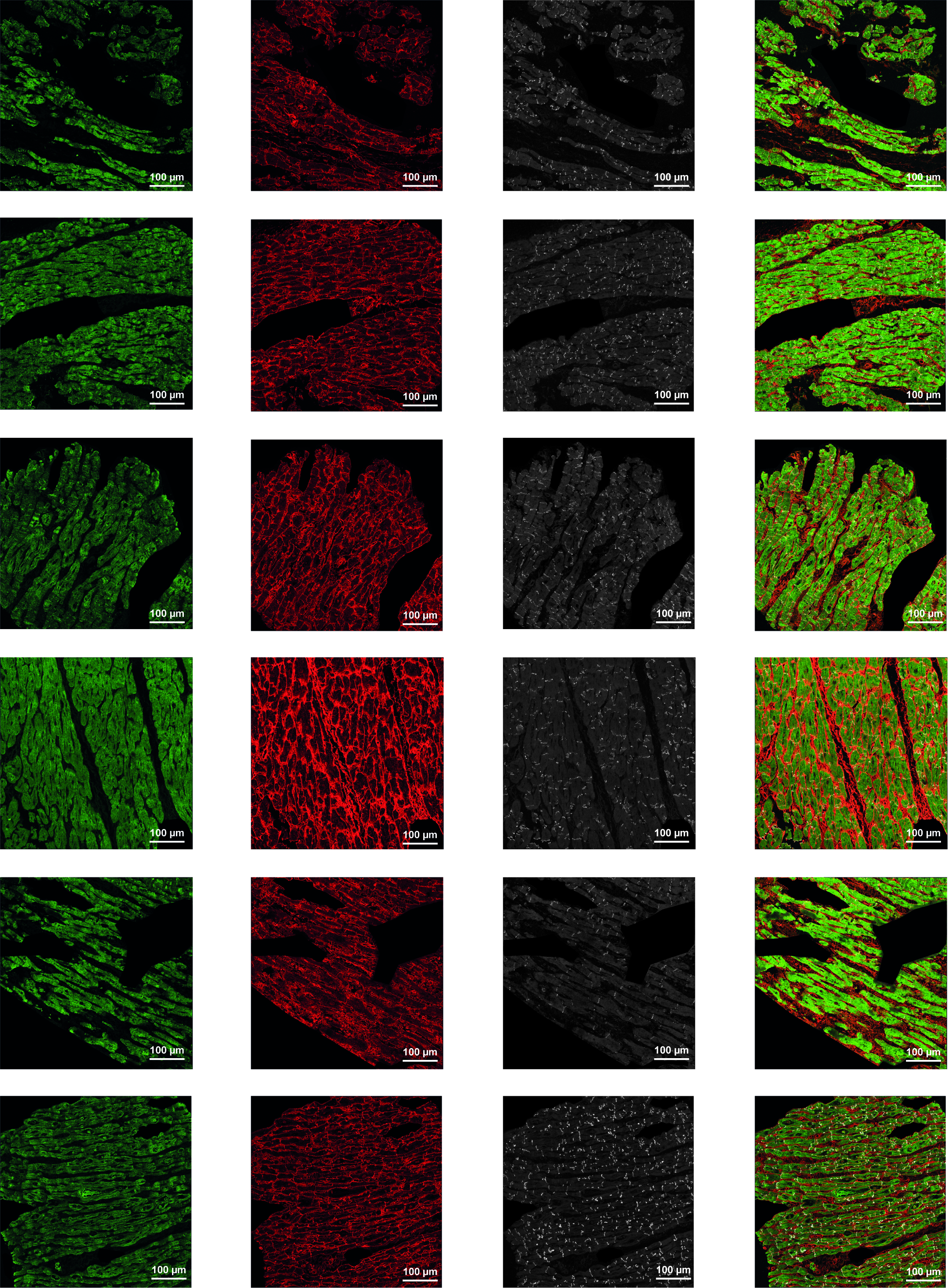

Supplement: Supplementary 1 — Fig. S1. Comparison of CX43 features between male and female donors of the same age range. Fig. S2. Comparison of the analysis of CX43 remodeling with respect to the cardiomyocyte area or the whole LV tissue. Fig. S3. Evaluation of conduction velocity for all simulated scenarios involving reduction in the longitudinal diffusion coefficient, increase in transverse-to-longitudinal diffusion ratio, increase in the content of fibrosis, and the combination of these 3 factors. Fig. S4. Repolarization gradient on epicardial meshes. Fig. S5. Images of the fluorescence immunohistochemistry of all the donors. Fig. S6. Methodology used for fibrosis quantification. Fig. S7. Images of picrosirius red histochemistry used to validate WGA-based fibrosis quantification method. Fig. S8. Validation of the WGA-based method of fibrosis quantification with picrosirius red staining. [file research.0254.f1.zip › Figure S5_6.tif]

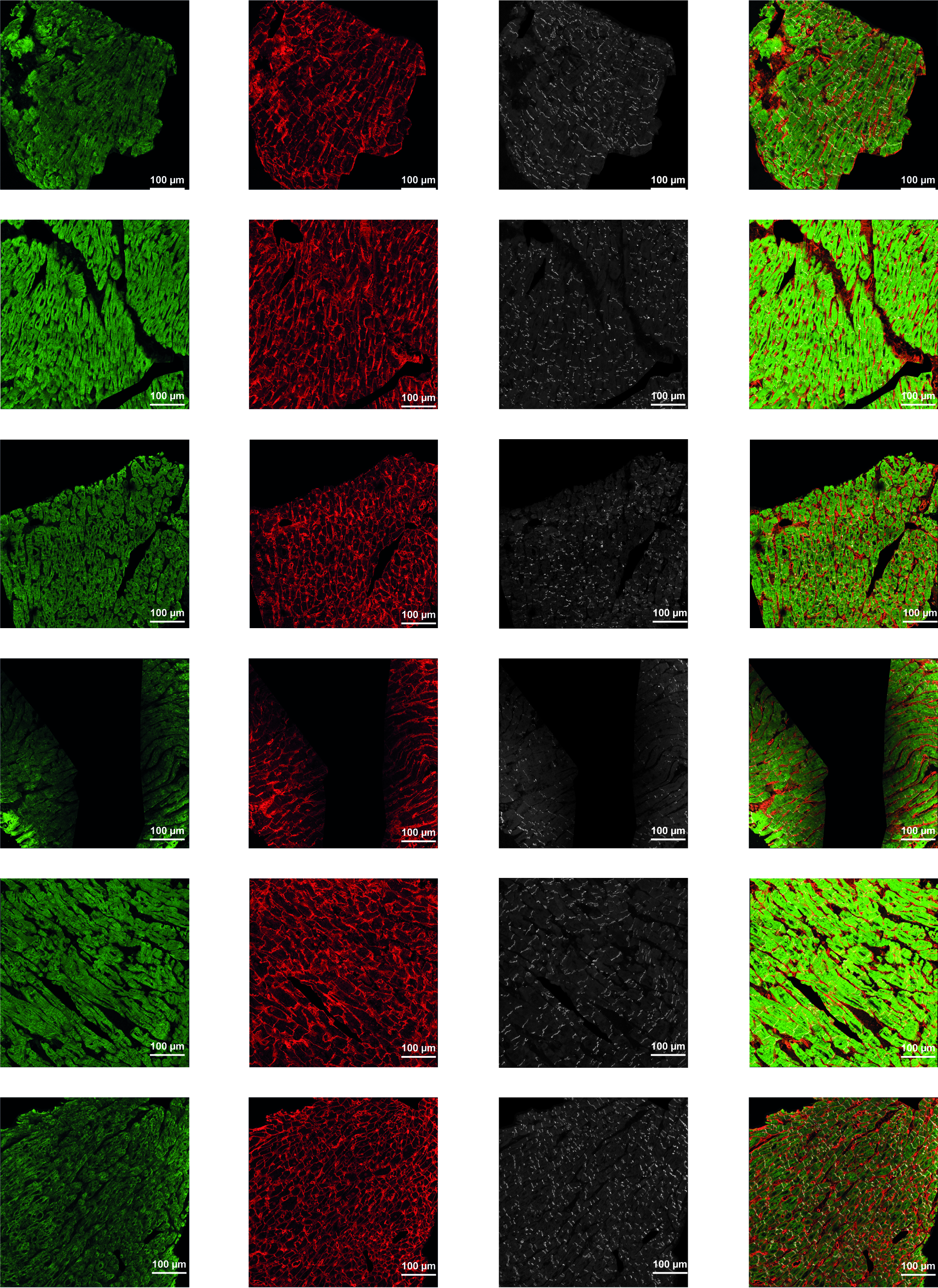

Supplement: Supplementary 1 — Fig. S1. Comparison of CX43 features between male and female donors of the same age range. Fig. S2. Comparison of the analysis of CX43 remodeling with respect to the cardiomyocyte area or the whole LV tissue. Fig. S3. Evaluation of conduction velocity for all simulated scenarios involving reduction in the longitudinal diffusion coefficient, increase in transverse-to-longitudinal diffusion ratio, increase in the content of fibrosis, and the combination of these 3 factors. Fig. S4. Repolarization gradient on epicardial meshes. Fig. S5. Images of the fluorescence immunohistochemistry of all the donors. Fig. S6. Methodology used for fibrosis quantification. Fig. S7. Images of picrosirius red histochemistry used to validate WGA-based fibrosis quantification method. Fig. S8. Validation of the WGA-based method of fibrosis quantification with picrosirius red staining. [file research.0254.f1.zip › Figure S5_7.tif]

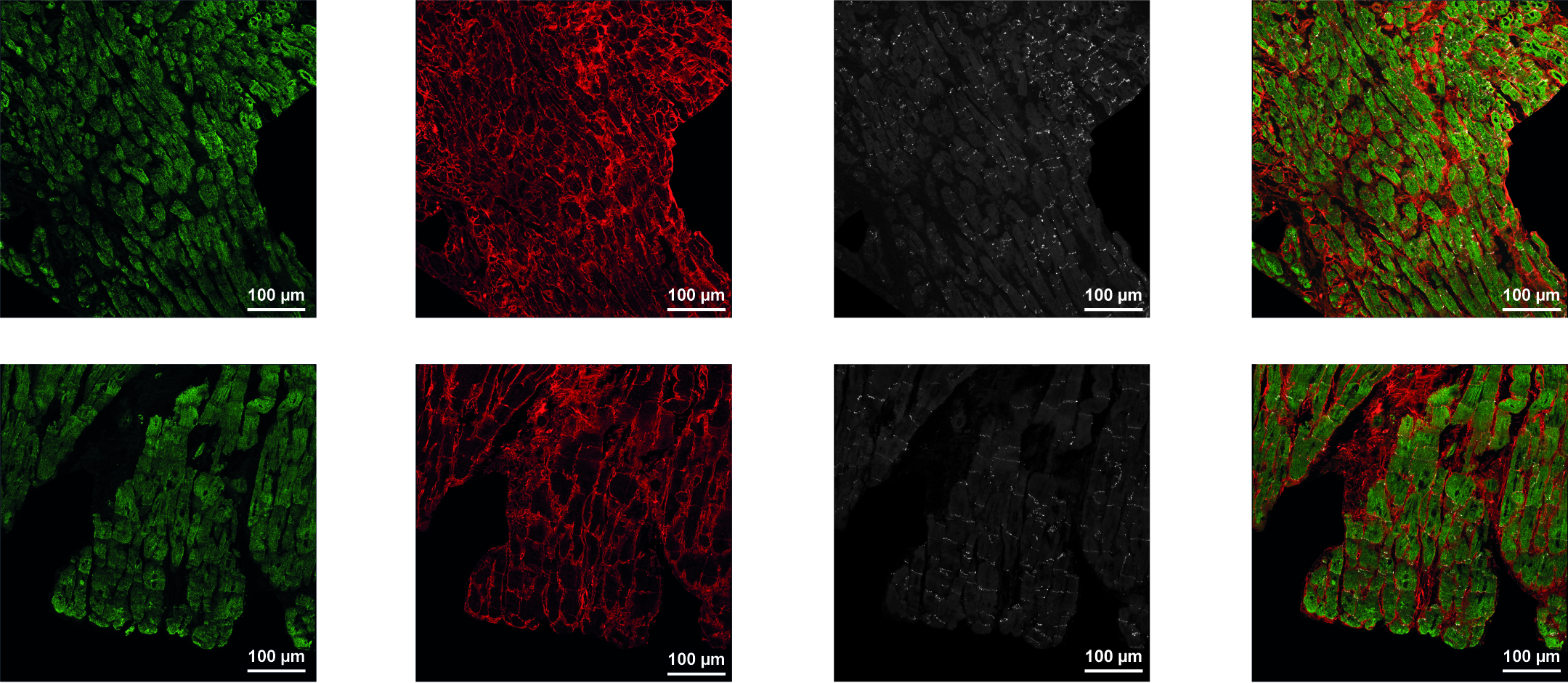

Supplement: Supplementary 1 — Fig. S1. Comparison of CX43 features between male and female donors of the same age range. Fig. S2. Comparison of the analysis of CX43 remodeling with respect to the cardiomyocyte area or the whole LV tissue. Fig. S3. Evaluation of conduction velocity for all simulated scenarios involving reduction in the longitudinal diffusion coefficient, increase in transverse-to-longitudinal diffusion ratio, increase in the content of fibrosis, and the combination of these 3 factors. Fig. S4. Repolarization gradient on epicardial meshes. Fig. S5. Images of the fluorescence immunohistochemistry of all the donors. Fig. S6. Methodology used for fibrosis quantification. Fig. S7. Images of picrosirius red histochemistry used to validate WGA-based fibrosis quantification method. Fig. S8. Validation of the WGA-based method of fibrosis quantification with picrosirius red staining. [file research.0254.f1.zip › Figure S5_8.tif]

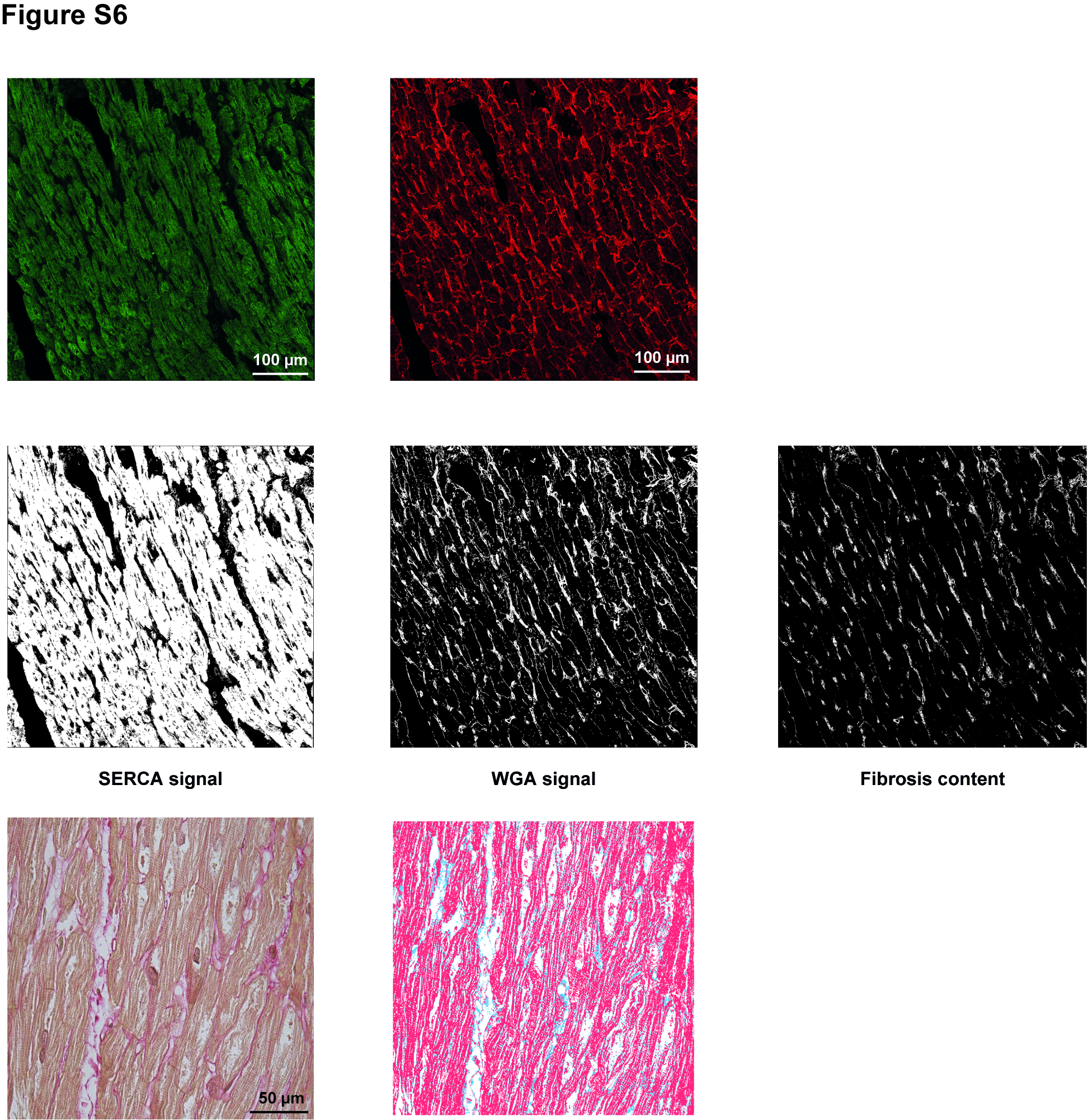

Supplement: Supplementary 1 — Fig. S1. Comparison of CX43 features between male and female donors of the same age range. Fig. S2. Comparison of the analysis of CX43 remodeling with respect to the cardiomyocyte area or the whole LV tissue. Fig. S3. Evaluation of conduction velocity for all simulated scenarios involving reduction in the longitudinal diffusion coefficient, increase in transverse-to-longitudinal diffusion ratio, increase in the content of fibrosis, and the combination of these 3 factors. Fig. S4. Repolarization gradient on epicardial meshes. Fig. S5. Images of the fluorescence immunohistochemistry of all the donors. Fig. S6. Methodology used for fibrosis quantification. Fig. S7. Images of picrosirius red histochemistry used to validate WGA-based fibrosis quantification method. Fig. S8. Validation of the WGA-based method of fibrosis quantification with picrosirius red staining. [file research.0254.f1.zip › Figure S6.tif]

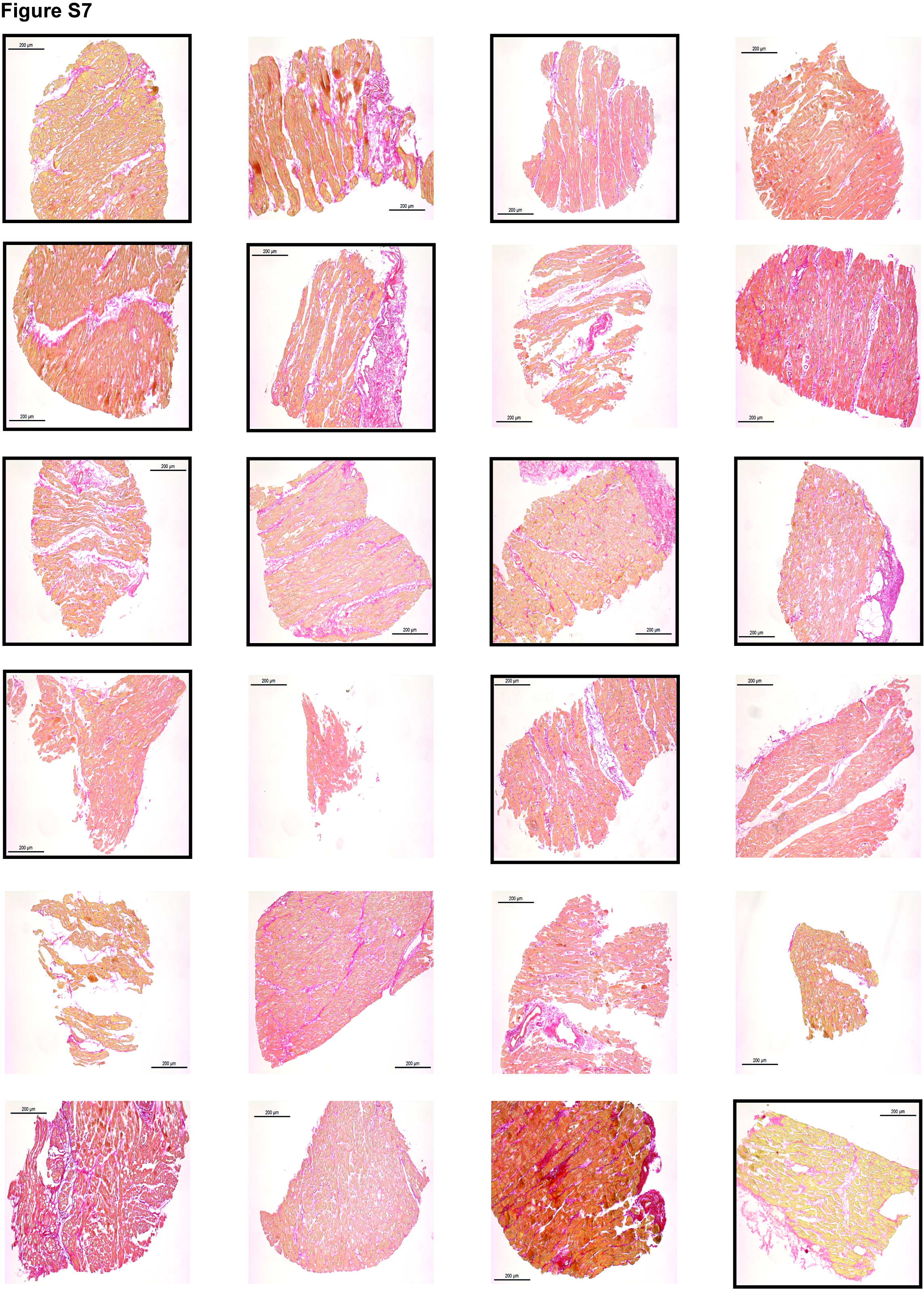

Supplement: Supplementary 1 — Fig. S1. Comparison of CX43 features between male and female donors of the same age range. Fig. S2. Comparison of the analysis of CX43 remodeling with respect to the cardiomyocyte area or the whole LV tissue. Fig. S3. Evaluation of conduction velocity for all simulated scenarios involving reduction in the longitudinal diffusion coefficient, increase in transverse-to-longitudinal diffusion ratio, increase in the content of fibrosis, and the combination of these 3 factors. Fig. S4. Repolarization gradient on epicardial meshes. Fig. S5. Images of the fluorescence immunohistochemistry of all the donors. Fig. S6. Methodology used for fibrosis quantification. Fig. S7. Images of picrosirius red histochemistry used to validate WGA-based fibrosis quantification method. Fig. S8. Validation of the WGA-based method of fibrosis quantification with picrosirius red staining. [file research.0254.f1.zip › Figure S7_1.tif]

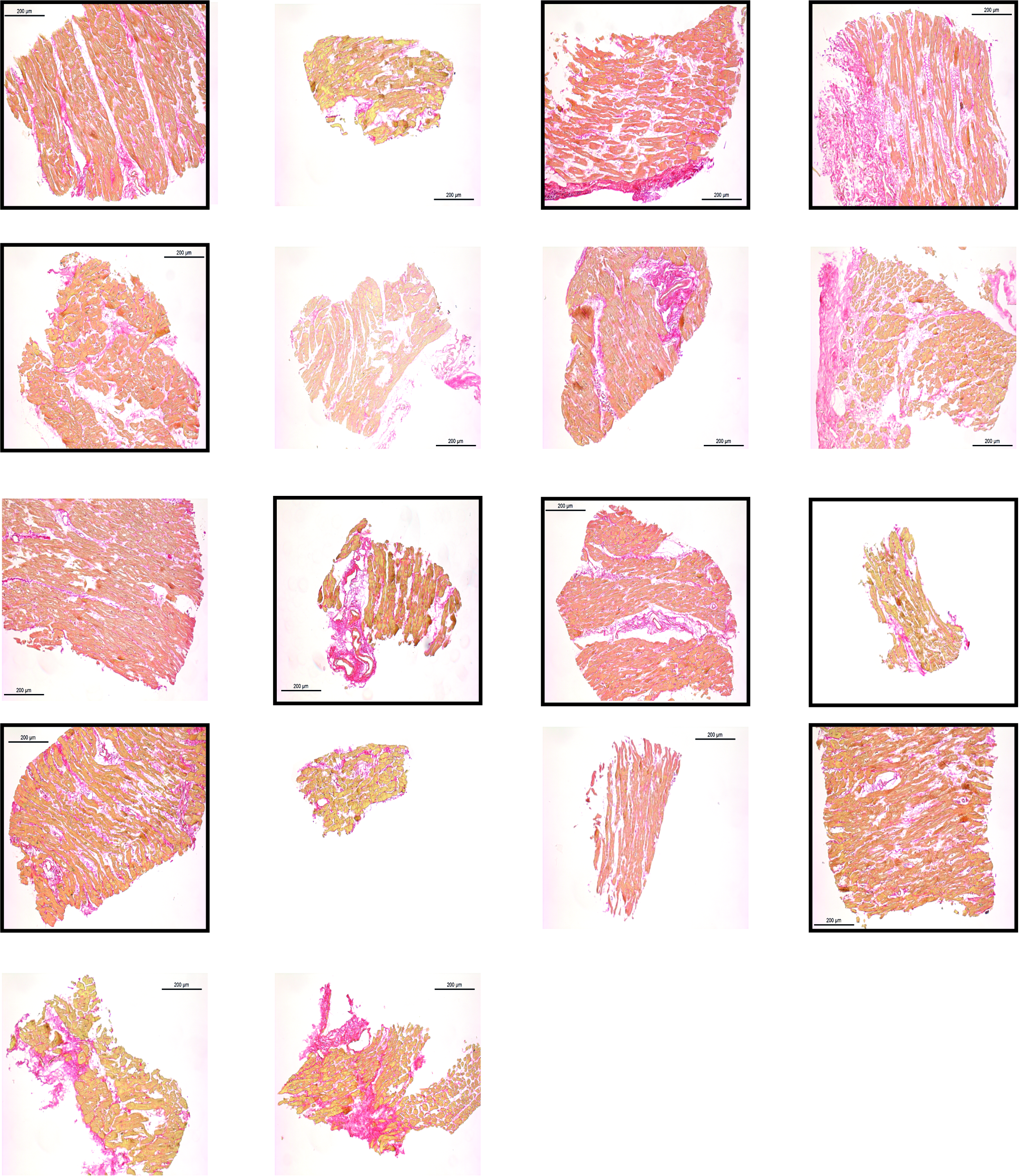

Supplement: Supplementary 1 — Fig. S1. Comparison of CX43 features between male and female donors of the same age range. Fig. S2. Comparison of the analysis of CX43 remodeling with respect to the cardiomyocyte area or the whole LV tissue. Fig. S3. Evaluation of conduction velocity for all simulated scenarios involving reduction in the longitudinal diffusion coefficient, increase in transverse-to-longitudinal diffusion ratio, increase in the content of fibrosis, and the combination of these 3 factors. Fig. S4. Repolarization gradient on epicardial meshes. Fig. S5. Images of the fluorescence immunohistochemistry of all the donors. Fig. S6. Methodology used for fibrosis quantification. Fig. S7. Images of picrosirius red histochemistry used to validate WGA-based fibrosis quantification method. Fig. S8. Validation of the WGA-based method of fibrosis quantification with picrosirius red staining. [file research.0254.f1.zip › Figure S7_2.tif]

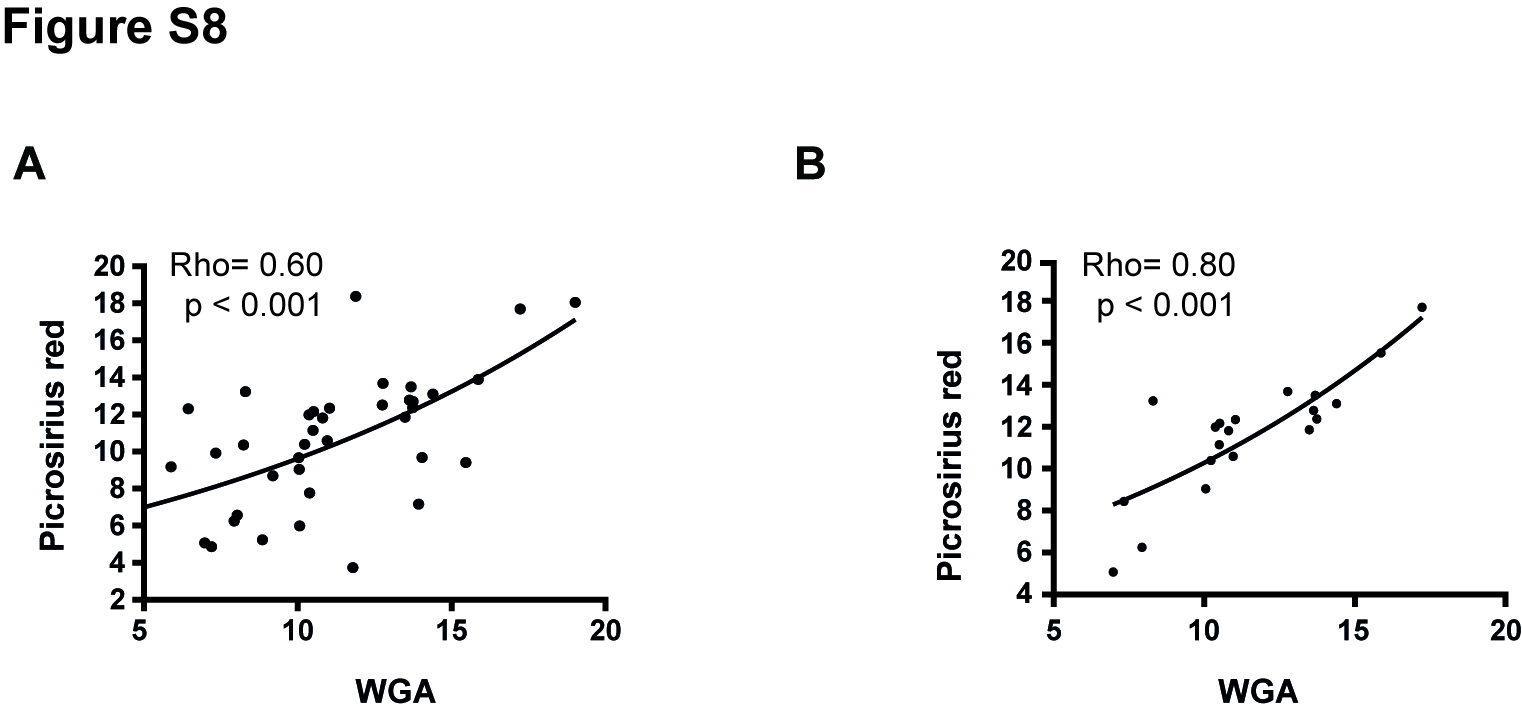

Supplement: Supplementary 1 — Fig. S1. Comparison of CX43 features between male and female donors of the same age range. Fig. S2. Comparison of the analysis of CX43 remodeling with respect to the cardiomyocyte area or the whole LV tissue. Fig. S3. Evaluation of conduction velocity for all simulated scenarios involving reduction in the longitudinal diffusion coefficient, increase in transverse-to-longitudinal diffusion ratio, increase in the content of fibrosis, and the combination of these 3 factors. Fig. S4. Repolarization gradient on epicardial meshes. Fig. S5. Images of the fluorescence immunohistochemistry of all the donors. Fig. S6. Methodology used for fibrosis quantification. Fig. S7. Images of picrosirius red histochemistry used to validate WGA-based fibrosis quantification method. Fig. S8. Validation of the WGA-based method of fibrosis quantification with picrosirius red staining. [file research.0254.f1.zip › Figure S8.tif]
